# Concordance in assessments between investigators and blinded independent central review (BICR) in hematology oncology clinical trials: a meta-analysis

**DOI:** 10.1093/oncolo/oyaf375

**Published:** 2025-11-09

**Authors:** Xiaoyu Tang, Yang Dang, Siying Han, Bohan Cui, Yi Kang, Xiaoyu Luo, Hui Zhang

**Affiliations:** Academy of Pharmacy, Xi’an Jiaotong-Liverpool University, Suzhou, Jiangsu, China; Institute of Population Health, University of Liverpool, Liverpool, United Kingdom; Oncology Clinical Development, Pfizer Inc, Beijing, China; Department of Biostatistics, Boston University, Boston, Massachusetts, United States; Academy of Pharmacy, Xi’an Jiaotong-Liverpool University, Suzhou, Jiangsu, China; Oncology Clinical Development, Pfizer Inc, Shanghai, China; Academy of Pharmacy, Xi’an Jiaotong-Liverpool University, Suzhou, Jiangsu, China; China Statistics, Pfizer Inc, Shanghai, China

**Keywords:** hematology, blinded independent central review, progression free survival, objective response rates, clinical trials, tumor assessments

## Abstract

**Background:**

Blinded independent central review (BICR) mitigates assessment bias in oncology trials but imposes significant operational burdens. Its value in hematologic malignancies—where multimodal response criteria reduce reliance on subjective imaging assessments compared to solid tumors—remains unestablished. This meta-analysis evaluates BICR-investigator concordance specifically in hematology trials.

**Methods:**

We systematically identified Phase II/III hematology trials (2014-2024) reporting progression-free survival (PFS) and/or objective response rate (ORR) assessments by both investigators and BICR from PubMed. Agreement was quantified using Pearson/Spearman correlation, pooled hazard ratio ratio (HRR, HR_INV_/HR_BICR_) for PFS, and odds ratio ratio for ORR (OddsRR, OR_INV_/OR_BICR_). We also analyzed the odds ratio for ORR for single arms (Odds_INV_/Odds_BICR_). Subgroup analyses assessed the impact of masking, cancer type based on imaging dependence, and sample size.

**Results:**

Data from 70 studies (37 PFS comparisons; 23 ORR comparisons; 29 single-arm ORR) were analyzed. For PFS, the pooled HRR was 0.96 (95% CI: 0.89, 1.03), with perfect agreement in statistical significance (Cohen’s kappa = 1). For ORR, the pooled OddsRR was 0.99 (95% CI: 0.85, 1.14). Single-arm trials showed minimal odds difference between assessors (OR = 1.02, 95% CI: 0.90, 1.17). Subgroup analyses (masking, cancer type, sample size) consistently showed high agreement.

**Conclusions:**

Investigator and BICR assessments demonstrated substantial concordance in hematology trials. The common applications of BICR in registration trials provide minimal added value for primary endpoint validation in this setting. We recommend prioritizing investigator training and standardized criteria to optimize resource allocation.

Implications for Practice Our research demonstrates high concordance between investigator and blinded independent central review (BICR) assessments for key endpoints, including progression-free survival and objective response rate, in hematologic malignancy trials. The findings challenge the common practice of BICR in pivotal hematology trials, as it offers minimal added value for primary endpoint validation. Our study provides evidence-based recommendation to reallocate the substantial resources and operational burden associated with BICR toward more impactful activities, such as enhanced investigator training and standardization of response criteria, thereby optimizing trial efficiency without compromising scientific integrity.

## Introduction

The evaluation of treatment response in oncology clinical trials relies on standardized criteria to ensure objective assessment of therapeutic efficacy. Blinded independent central review (BICR)—a process wherein experts masked to treatment assignment evaluate radiographic or clinical data—was developed to mitigate potential bias in investigator-based assessments, particularly in open-label trials where treatment knowledge may influence progression determinations.^1^^,^^2^ Regulatory agencies frequently mandate BICR for pivotal trials to support endpoint reliability,^3^^,^^4^ though its added value remains contentious given substantial resource demands and operational complexities.^5–7^

Recent meta-analyses have systematically evaluated BICR-investigator concordance patterns, primarily in solid tumors or mixed cohorts including both solid tumors and hematologic malignancies. Foundational work by Amit et al. demonstrated negligible difference in PFS assessments across 36 trials.^8^ This high concordance was confirmed by Russo et al. in 28 Phase III trials,^4^ where no significant hazard ratio differences emerged, and further supported by Jacobs et al. in metastatic breast cancer.^9^ While Zhang et al. observed no systematic bias in Phase III trials, they noted statistically discordant inferences in a subset of assessments.^10^ Even though D’Ambrosio et al. showed systematic overestimation of PFS by investigators in immunotherapy trials and Lian et al. observed this in open-label trials for the mixed cohort, the discrepancies were numerically small.^5^^,^^11^ Zettler et al. found no evidence of evaluation bias in the assessment of ORR among pivotal trials supporting recent FDA approvals of anticancer agents for solid tumor indications.^12^

In solid tumors, response assessments rely exclusively on predefined quantitative imaging criteria such as Response Evaluation Criteria in Solid Tumors (RECIST v1.1).^13^ These evaluations require subjective interpretation during lesion selection, measurement, and identification of new lesions—introducing inherent variability and potential assessment bias.^5^ Unlike solid tumors, hematologic malignancies employ multimodal assessment frameworks with reduced reliance on imaging for some indications, potentially reducing evaluation bias. For example, lymphoma follows Lugano criteria combining PET-CT imaging and histopathology,^14^ but multiple myeloma applies IMWG standards where most of response assessments derive from serum/urine parameters, especially in patients with no measurable extramedullary diseases (EMD) at baseline.^15^ This reliance on non-imaging data sources—particularly for acute leukemias and myeloma where clinical and laboratory parameters dominate—substantially reduces the subjectivity inherent in radiographic interpretation. Despite this potential bias reduction, hematology trials are predominantly open-label due to distinctive toxicity profiles and special administration routes. In such designs, knowledge of treatment assignment may introduce investigator bias.

To our knowledge, the concordance has not been systematically evaluated for hematologic malignancies, but only in individual trial comparisons.^16^ To address this gap, we conducted the first systematic evaluation of BICR-investigator concordance across Phase II/III hematology trials. We quantified agreement for progression-free survival (PFS) and objective response rate (ORR)—common primary endpoints in Phase II/III oncology trials^17^—assessing both treatment effect estimates and statistical inferences to evaluate BICR’s added value in hematologic malignancies. The protocol was registered in the PROSPERO database (ID CRD420251104087).

## Methods

### Searching strategy and selection criteria

According to the Preferred Reporting Items for Systematic Reviews and Meta-Analyses (PRISMA) guidance, we searched PubMed for publications of phase II and phase III hematology clinical trials from January 1, 2014, to November 27, 2024, using both Medical Subject Headings (MeSH) terms and free-text words (Text S1). Eligible studies included phase II or III hematology clinical trials that directly evaluated therapeutic efficacy of anticancer treatments for hematologic malignancies, with tumor response and progression assessments conducted by both investigators and BICR, and summary statistics from both assessors reported (eg, hazard ratios for PFS, proportions/cases of overall responses). While single-arm trials (SATs) and randomized controlled trials (RCTs) have distinct objectives, SATs were included in our study due to their established role in supporting regulatory accelerated approvals in hematologic malignancies, particularly in relapsed/refractory settings.^17^^,^^18^ Similarly, the ORR was included as it is a common primary endpoint in SATs. In addition, it is allowed to be used as an early endpoint in RCTs to support accelerated approval.^17^^,^^18^ We excluded subgroup analyses, follow-up analyses, and studies with fewer than 10 participants. In addition, some multi-arm RCTs compared multiple treatments A, B, against a common control C, and we considered A vs. C and B vs. C as independent comparisons. Three authors (BC, XL, and YK) independently screened titles/abstracts to assess eligibility. Full-text articles were obtained for studies meeting initial inclusion criteria. Final inclusion was determined through full-text review against eligibility criteria. Disagreements were resolved through discussion with the other team members (XT, DY, SH, YK, and HZ).

### Data extraction

Data extraction was conducted independently by 3 authors (BC, XL, and YK) using a standardized form and subsequently validated by 2 additional authors (SH and XT). Any discrepancies were resolved through team consensus (XT, DY, and HZ). For each included study, the following variables were extracted: study identifiers (title, first author, publication year, National Clinical Trial [NCT] number); trial characteristics (phase, masking [open-label vs. double-blind], total sample size, sample size per treatment group [in randomized trials], cancer type, medical therapy setting, response assessment criteria, primary endpoint[s], names of treatments); and summary statistics for outcomes data, including HRs for PFSs with 95% confidence intervals (CIs) as assessed by both investigators and BICR, ORR with 95% CIs, and the number of responses (for both treatment and control groups in randomized trials, and for the single group in single-arm trials). For trials where PFS was the primary endpoint (assessed by investigators or BICR), the corresponding HR *P*-values and statistical significance were also extracted. Study characteristics are detailed in Table S1.

### Statistical analysis

#### PFS analysis

The correlation between the logarithm of the hazard ratio for PFS assessed by investigators (log($HR_{\mathrm{INV}})$) and the logarithm of the hazard ratio assessed by BICR (log($HR_{\mathrm{BICR}})$) was evaluated using Pearson’s correlation coefficient when normality assumptions were met based on the Shapiro-Wilk test^19^; otherwise, Spearman’s correlation was employed. Subsequently, we performed a meta-analysis using a fixed-effects model with inverse-variance weighting to estimate the pooled hazard ratio ratio (HRR), defined as:


$$\mathrm{HRR}=\frac{HR_{\mathrm{INV}}}{HR_{\mathrm{BICR}}}$$

An HRR < 1 indicates that $HR_{\mathrm{inv}}$ is smaller than $HR_{\mathrm{BICR}}$, suggesting a more favorable outcome for the experimental group compared to the control group based on investigator assessments. The meta-analysis was conducted on log(HRR), and the corresponding standard error was derived from the standard errors of log($HR_{\mathrm{BICR}})$ and log($HR_{\mathrm{INV}}).$  ^20^ If significant statistical heterogeneity was found (the *P* value for Cochran’s Q test was less than 0.05 or the $I^{2}$ was over 50%), a random-effects model would be adopted to take the heterogeneity into account. Subgroup analyses were conducted based on masking status (open-label vs. blinded), sample size, and cancer type. For cancer type, subgroups comprised indications requiring imaging for response assessment (eg, lymphoma and chronic lymphocytic leukemia) and indications not primarily reliant on imaging (eg, acute leukemia and multiple myeloma). The correlations were not calculated if number of studies was less than 5, and the meta-analyses were not conducted if number of studies was less than 3.

Additionally, for trials where PFS (assessed by either investigators or BICR) was the primary endpoint and the treatment comparison was formally tested against a pre-defined α level, we evaluated the agreement in statistical significance between investigator and BICR assessments. The consistency in statistical inference (significant vs. non-significant) was quantified using Cohen’s kappa coefficient.

#### ORR analysis

To account for the different trial designs, separate analytical approaches were conducted for RCTs and SATs. For 2-arm trials, we analyzed the correlation between the logarithm of the odds ratio for objective response assessed by investigators (log($OR_{\mathrm{INV}})$) and the logarithm of the odds ratio assessed by BICR (log($OR_{\mathrm{BICR}})$). We performed a meta-analysis to estimate the pooled odds ratio ratio (OddsRR), defined as:


$$\mathrm{OddsRR}=\frac{OR_{\mathrm{INV}}}{OR_{\mathrm{BICR}}}=\frac{\mathrm{Odd}s_{\mathrm{INV}}^{\mathrm{trt}}/\mathrm{Odd}s_{\mathrm{INV}}^{\mathrm{control}}}{\mathrm{Odd}s_{\mathrm{BICR}}^{\mathrm{trt}}/\mathrm{Odd}s_{\mathrm{BICR}}^{\mathrm{control}}}$$

where odds of response equals to ORR/(1-ORR). Hence, an OddsRR > 1 indicates that $OR_{\mathrm{INV}}$ is larger than $OR_{\mathrm{BICR}}$, suggesting a more favorable outcome for the experimental group compared to the control group based on investigator assessment. The methods for conducting correlation analyses, meta-analyses and subgroup analyses are analogous to those for PFS described in “ PFS analysis.”

For single-arm trials with ORR as an endpoint (where OddsRR is not estimable without a control group), we calculated odds ratio of response between investigator and BICR assessment, which is defined as:


$$OR=\frac{\mathrm{Odd}s_{\mathrm{INV}}}{\mathrm{Odd}s_{\mathrm{BICR}}}$$

We synthesized the logarithm of the treatment-group odds ratio (log ($OR_{\;\text{exp}\;}))$ in single-arm trials. In addition, we separately pooled log ($OR_{\;\text{exp}\;})$ and log($OR_{\mathrm{control}})$ for 2-arm trials.

#### Reporting bias and risk of bias

To assess reporting bias, we summarized the number and proportion of studies that stated they performed both investigator and BICR assessments but failed to report data from one of these assessments. In such cases, the absence of BICR or investigator-assessed data may be due to discordance between assessments, potentially introducing reporting bias.

The risk of bias for included randomized studies was assessed independently by 2 authors (BC and SH) using the Cochrane Risk of Bias tool version 2 (RoB-2).^21^ Discrepancies were resolved through discussion with other team members. Assessments were classified as “Low risk,” “High risk,” or “Some concerns” across the following domains: randomization process, deviations from intended interventions, missing outcome data, measurement of the outcome, and selection of the reported result.

## Results

**Figure 1.**
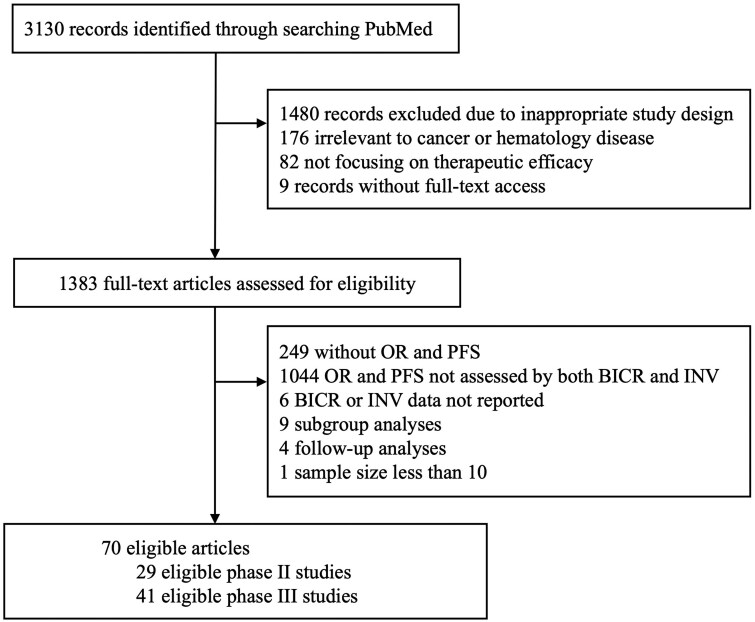
Flow chart of study selection.

We identified 70 eligible studies in our systematic search (Figure 1, Table S1). Because one of the trials has 2 experimental arms and a control arm, we treated the comparison between each experimental arm and the control arm as independent comparison in our analysis. In addition, we treated one trial only reporting results for 2 subgroups as 2 independent comparisons. The summary statistics of eligible comparisons of PFS analysis and ORR analysis were reported in Table 1. We included 37 comparisons for PFS analysis, 23 comparisons for 2-arm ORR analysis. Most of comparisons are open-label studies (78% in PFS comparisons and 91% in ORR comparisons), and from Phase III clinical trials (95% in PFS comparisons and 91% in ORR comparisons). In addition, we have 29 studies for single-arm ORR analysis.

**Table 1.** Descriptive summaries of characteristics.

Characteristics	PFS comparisons (n = 37)	ORR comparisons (n = 23)	Single-arm comparisons (n = 29)
**Masking**			
** Open-label**	29 (78.4%)	21 (91.3%)	29 (100%)
** Blinded**	8 (21.6%)	2 (8.7%)	0
**Phase**			
** Phase II**	2 (5.4%)	2 (8.7%)	27 (93.1%)
** Phase III**	35 (94.6%)	21 (91.3%)	2 (6.9%)
**Sample size**			
** ≤250**	6 (16.2%)	5 (21.7%)	29 (100%)
** 250-350**	9 (24.3%)	3 (13.0%)	0
** 350-450**	9 (24.3%)	8 (34.8%)	0
** >450**	13 (35.1%)	7 (30.4%)	0
**Cancer type**			
** Lymphoma**			
** cHL**	2 (5.4%)	0	5 (17.2%)
** iNHL**	1 (2.7%)	1 (4.3%)	1 (3.4%)
** FL**	1 (2.7%)	2 (8.7%)	5 (17.2%)
** MCL**	2 (5.4%)	2 (8.7%)	1 (3.4%)
** CTCL**	1 (2.7%)	1 (4.3%)	0
** PTCL**	2 (5.4%)	0	4 (13.8%)
** DLBCL**	3 (6.1%)	1 (4.3%)	2 (6.9%)
** ENKTL**	0	0	1 (3.4%)
** LBCL**	0	0	2 (6.9%)
** WM**	1 (2.7%)	0	0
** MZL**	0	0	2 (6.9%)
** TFHL**	1 (2.7%)	0	0
** FL/MZL**	1 (2.7%)	1 (4.3%)	0
** DLBCL/FL**	1 (2.7%)	0	0
** Leukemia**			
** CLL**	9 (24.3%)	7 (30.4%)	0
** ALL**	0	1 (4.3%)	0
** Myeloma**			
** MM**	6 (16.2%)	3 (13.0%)	3 (10.3%)
** Leukemia/Lymphoma**			
** CLL/SLL**	5 (13.5%)	3 (13.0%)	2 (7.4%)
** T-cell Leu/Lym**	0	0	1 (3.4%)
** Myeloid**			
** MDS**	1 (2.7%)	1 (4.3%)	0

n: number of studies.

Abbreviations: ALL, Acute Lymphoblastic Leukemia; cHL, Classical Hodgkin Lymphoma; CLL, Chronic Lymphocytic Leukemia; CTCL, Cutaneous T-Cell Lymphoma; DLBCL, Diffuse Large B-Cell Lymphoma; ENKTL, Extranodal Natural Killer/T-Cell Lymphoma; FL, Follicular Lymphoma; iNHL, Indolent NonHodgkin Lymphoma; LBCL, Large B-Cell Lymphoma; MCL, Mantle Cell Lymphoma; MDS, Myelodysplastic Syndromes; MM, Multiple Myeloma; MZL, Marginal Zone Lymphoma; ORR, objective response rate; PFS, progression-free survival; PTCL, Peripheral T-Cell Lymphoma; T-cell Leu/Lym, T-Cell Leukemia/Lymphoma; WM, Waldenström Macroglobulinemia.

### Agreements in HR for PFS

The correlations and results from meta-analysis were summarized in Table 2. Among 37 PFS comparisons, we observed strong correlations between log($HR_{\mathrm{INV}})$ and log($HR_{\mathrm{BICR}})$ in both the overall analysis and all subgroups, with all point estimates exceeding 0.95 and lower confidence interval bounds above 0.78. Fixed-effects model was employed to conduct meta-analyses. The pooled HRR was 0.96 (95% CI: 0.89, 1.03), which was close to 1 and indicated no statistically significant difference between $HR_{\mathrm{BICR}}$ and $HR_{\mathrm{INV}}$ (Figure S1). In subgroup analyses, though open-label trials showed a pooled HRR slightly below 1 while blinded trials showed a point estimate slightly above 1, neither reached statistical significance (Figure S2). In indications where image assessment only required for partial patients (EMD) for disease assessment, the subgroup analysis demonstrated a HRR point estimate of 1.00 (95% CI: 0.86,1.16). The HRR estimate in indications where imaging required for all patients was 0.95 (95% CI: 0.87, 1.03). Both estimates approximated 1 and were non-significant (Figure S3). Similarly, HRR were comparable between trials with sample sizes ≤350  versus those with >350 patients, with both estimates being non-significant (Figure S4).

**Table 2.** Agreement assessment of PFS between BICR and investigators.

Characteristics		Correlation r (95%CI)		HRR (95%CI)	
		n	Value	n	Value
**Overall**		37	0.97* (0.91, 0.99)	35	0.96 (0.89, 1.03)
**Masking**					
	**Open label**	29	0.96* (0.87, 0.99)	27	0.95 (0.87, 1.03)
	**Blinded**	8	0.99 (0.97, 1.00)	8	1.01 (0.87, 1.18)
**Cancer Types**					
	**Imaging-required for all patients^a^**	30	0.97* (0.89, 0.99)	29	0.95 (0.87, 1.03)
	**Imaging-required for partial patients^b^**	7	0.98 (0.89, 1.00)	6	1.00 (0.86, 1.16)
**Sample Size**					
	**Sample size ≤ 350**	15	0.95 (0.86, 0.98)	13	0.95 (0.81, 1.11)
	**Sample size > 350**	22	0.96* (0.78, 0.99)	22	0.96 (0.89, 1.05)

n: number of studies.

* Spearman correlation was used due to rejection of normality assumption based on the Shapiro-Wilk test.

a Indications including LYM, CLL/SLL, CLL.

b Indications including MM, MDS, LEU except CLL.

Note: HRR standard errors could not be estimated for 2 studies due to missing confidence intervals and SE for HRs. Both studies were open-label trials; one study involved a fully image-dependent cancer type and the other a partially image-dependent type; both studies had sample sizes ≤350.

**Figure 2.**
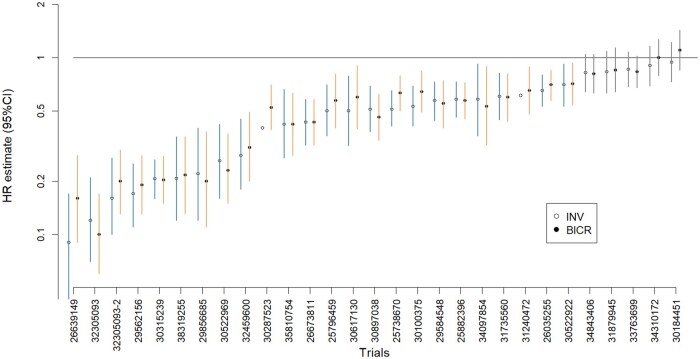
HR estimates and 95% confidence intervals by investigator and BICR assessments. Note: The 2 points without error bars have reported significant p-values but no confidence intervals.

Among 33 comparisons with PFS as the primary endpoint, investigator and BICR assessments showed perfect concordance in statistical significance determinations: 26 comparisons were statistically significant by both assessments and 7 were non-significant by both assessments (Table S2, Figure 2), resulting in a Cohen’s kappa coefficient of 1. While no discordance in statistical significance was observed, small differences in point estimates could theoretically alter trial conclusions when the treatment effect is marginal (ie, HR close to 1) or the sample size is small (where the CI might be wide due to large variation). This could occur if such differences shift the upper confidence limit sufficiently to lead one assessment method’s CI to cross the significance threshold while the other’s does not.

### Agreements in ORR

Table 3 presents the meta-analysis results and correlations for OddsRR analysis. Among 23 ORR comparisons, strong correlations between log(O$R_{\mathrm{INV}})$ and log($OR_{\mathrm{BICR}})$ were observed in both the overall analysis and all subgroups. However, in the subgroup with a sample size ≤350, the confidence interval for the correlation was wide due to the small number of studies available. Among 23 two-arm trials, both the experimental group and the control group demonstrated significantly higher response rates when assessed by investigators versus BICR ($OR_{\;\text{exp}\;}$ = 1.23,95% CI: 1.02, 1.48; $OR_{\mathrm{control}}$ =1.30, 95% CI: 1.09, 1.55) (Table S3, Figures S5 and S6). However, the pooled OddsRR was 0.99 (95% CI: 0.85, 1.14), which was close to 1 and indicated no statistically significant difference between assessors for the treatment effect estimates (Figure S7). The combined OddsRRs in all other subgroups were also close to 1 and not significant (Figures S8-S10). Among 29 single-arm trials, the pooled $OR_{\mathrm{trt}}$ was 1.02 (95% CI: 0.90, 1.17), indicating a minimal and non-significant difference between odds of response assessed by investigators and BICR (Figure S11).

**Table 3.** Agreement assessment of ORR between BICR and INV.

Characteristics		Correlation r (95%CI)		ORR (95%CI)	
		n	Value	n	Value
**Overall**		23	0.92 (0.83, 0.97)	23	0.99 (0.85, 1.14)
**Masking**					
	**Open label**	21	0.92 (0.81, 0.97)	21	1.01 (0.87, 1.18)
	**Blinded**	2	NA	2	NA
**Caner Types**					
	**Imaging-required for all patients** ^a^	18	0.90 (0.76, 0.96)	18	0.99 (0.84, 1.18)
	**Imaging-required for partial patients** ^b^	5	0.98 (0.72, 1.00)	5	0.97 (0.73, 1.30)
**Sample Size**					
	**Sample size ≤ 350**	8	0.91 (0.55, 0.98)	8	0.91 (0.67, 1.24)
	**Sample size > 350**	15	0.94 (0.82, 0.98)	15	1.01 (0.86, 1.20)

n: number of studies.

a Indications including LYM, CLL/SLL, CLL.

b Indications including MM, MDS, LEU except CLL.

### Assessments of bias

In our analysis, 6 of 76 trials (7.8%) were excluded due to missing BICR or investigator assessment data, despite reporting that both evaluations were conducted. Given this small proportion, the exclusions are unlikely to have substantial impact on the results.

We have 42 randomized studies in total. Using the ROB-2 tool for risk of bias assessment, we found low risk of bias in 15 (36%) of studies and some concerns in 27 (64%) of studies; no comparisons were rated high risk (Table S4, Figure S12). Most concerns arose in open-label studies, where knowledge of treatment assignment could introduce bias in outcome assessments.

## Discussion

This meta-analysis represents the first systematic evaluation of concordance between investigator and BICR assessments in hematology oncology clinical trials. Our findings demonstrated high agreement in PFS and ORR assessments, with negligible differences in hazard ratio ratios (HRR) for PFS and OddsRR for ORR. Specifically, the pooled HRR of 0.96 (95% CI: 0.89, 1.03) indicates minimal systematic bias in PFS evaluations, with perfect agreement in statistical inferences (Cohen’s kappa = 1) which further supports the reliability of investigator assessments. Despite perfect agreement in statistical significance, the small differences in point estimates we observed could theoretically lead to different trial conclusions for treatments with a marginal treatment effect or in small studies with large variations. For ORR in 2-arm trials, despite this study observed investigators might tend to report more responses than BICR in both treatment and control arms, the impact on treatment effect estimates was negligible, which is shown by the pooled OddsRR of 0.99 (95% CI: 0.85, 1.14). In addition, the analysis in single arm-trials didn’t reveal the same level of favorable trend in investigator’s assessment: the pooled $OR_{\mathrm{trt}}$ of 1.02 (95% CI: 0.90, 1.17) confirms the strong concordance.

Unlike solid tumors assessed solely based on imaging (eg, RECIST criteria),^13^ hematologic malignancies employ multimodal frameworks integrating laboratory parameters, histopathology, and clinical findings. Meanwhile, in confirming progressive disease, laboratory parameters or histopathology results often deteriorate prior to EMD progression, as observed in multiple myeloma or acute leukemias. Therefore, progressive disease is typically assessed through objective parameters, but not imaging. This different feature likely reduces subjectivity in disease assessments compared to those relying solely on the imaging evaluation, explaining the high BICR-Investigator concordance observed in hematologic malignancies. The study results confirmed this by demonstrating negligible discrepancies in both PFS and ORR assessments. Furthermore, although disease assessments in hematologic malignancies all required multimodal frameworks, the level of dependency on imaging is different. The estimates for indications where imaging is only required for partial patients showed the level of bias may be even reduced compared with those indications where imaging is required for all patients. This may further indicate that the concern of subjective bias may be reduced with the reduced level of dependency on imaging evaluation. Despite most of hematology trials were open-label, investigator and BICR assessments are highly consistent, suggesting that standardized response criteria mitigate bias even when treatment assignments are known. In addition, the agreement was maintained across difference sample sizes.

The high concordance demonstrated in this meta-analysis challenges the necessity of BICR for all patients in hematology oncology trials. The conventional rationale for BICR rests on its high reliability, achieved through blinding to mitigate assessment bias. However, this specific strength is counterbalanced by significant limitations: informative censoring occurs when disease assessments cease or new anti-cancer therapies were initiated following investigator-reported progression, potentially skewing BICR-assessed survival estimates.^1^^,^^8^^,^^22–25^ Furthermore, in some cases, BICR evaluations lack the comprehensive clinical context available to investigators.^5^ Investigators typically account for clinical factors and conduct comprehensive assessments. For instance, when imaging findings are equivocal but a patient demonstrates clinically stable or improved status relative to baseline, investigators are more likely to assess stable disease; conversely, clinical deterioration may justify progressive disease despite ambiguous imaging. In this case, this integrated approach provides a more accurate reflection of treatment efficacy, whereas BICR lacks access to patients’ clinical information. This clinical context is often essential for accurate assessment, meaning that investigator evaluations can offer a superior level of clinical validity. In addition, our findings demonstrate that the reliability of investigator assessments in this field is higher than traditionally assumed, as evidenced by the minimal discrepancy in treatment effect estimates between review methods. Consequently, the marginal benefit of BICR's reliability is offset by its operational burdens and methodological constraints, while the high validity and demonstrated concordance of investigator assessments support their use as the primary source for endpoint validation.

Hence, we recommend a more risk-based and resource-efficient approach for central review. For most hematology trials, particularly those using well-established and multimodal response criteria, BICR could be omitted without compromising validity. However, for high-risk scenarios where the potential for assessment bias or unconventional response patterns increases, such as trials of novel therapeutic classes with the risk of atypical response patterns (eg, immunotherapy with potential for pseudo-progression), trials where the primary endpoint remains highly subjective, or trials in controversial or high-stakes therapeutic areas where regulatory scrutiny is anticipated to be intense, a random sample-based BICR auditing approach could be used for clinical trial quality control, as indicated by FDA guidance.^17^ Implementation needs a prespecified auditing plan detailing strategies for detecting potential assessment bias and mitigation plans, a process requiring discussions among health authority, sponsors, and investigators. A full BICR may be reserved to provide a supplementary analysis of the treatment effect if a significant discrepancy rate is found in the audit. The net effect of this change is a substantial overall reduction in resource expenditure across the clinical trial portfolio.

While similar conclusions were drawn from previous solid tumor meta-analyses, the practice of BICR has persisted, likely due to regulatory conservatism and sponsor risk aversion. However, the rationale for BICR is fundamentally weaker in hematologic malignancies due to the reduced reliance on subjective imaging. Moreover, unlike in solid tumors, the operational infrastructure for BICR is less matured in hematology malignancies. Our study provides the first comprehensive and disease-specific evidence to justify a re-evaluation of this practice within hematology oncology, providing a foundation for refining clinical trial standards within this specialty.

Our study has several limitations. First, subgroup analyses—particularly for ORR comparisons in blinded studies and indications depending on imaging for partial patients—were constrained by small sample sizes. This limited our ability to robustly explore assessment patterns across different masking methodologies or specific hematologic malignancies. The limited number of blinded studies is due to distinctive drug toxicities and administration routes. Future meta-analyses should prioritize expanding cancer types to clarify disease-specific patterns. Furthermore, the independence of central review may be compromised in trials where BICR only confirms investigator-assessed progression, potentially inflating concordance. Finally, our analysis focused on aggregate trial-level treatment effects because the lack of individual patient data precluded evaluation of concordance at the patient level, such as the exact timing of progression or response status for each patient. The individual-level data can provide deeper insights into the specific causes of the small differences we observed at the trial level.

In conclusion, BICR offers limited added value in hematology oncology trials given the high concordance with investigator assessments. To optimize resource allocation, we recommend conducting central review for pivotal trials in high-risk contexts as quality control tool or supplementary analyses, storing baseline and progression images alongside clinical data to enable ad hoc review, and optimizing investigator training and response criteria standardization to further minimize assessment discrepancies. Future efforts should refine these strategies to accelerate therapeutic development while maintaining rigorous endpoint validity.

## Supplementary Material

oyaf375_Supplementary_Data

## Data Availability

The data underlying this article are available in the article and in its online supplementary material.
