# Supplementary material for: Concordance in assessments between investigators and blinded independent central review (BICR) in hematology oncology clinical trials: a meta-analysis
Source: Oncologist. 2025 Nov 9;30(11):oyaf375. doi: 10.1093/oncolo/oyaf375 (PMC12622372; doi:10.1093/oncolo/oyaf375)

Figure S1. Forest plot of meta-analysis for HRR.


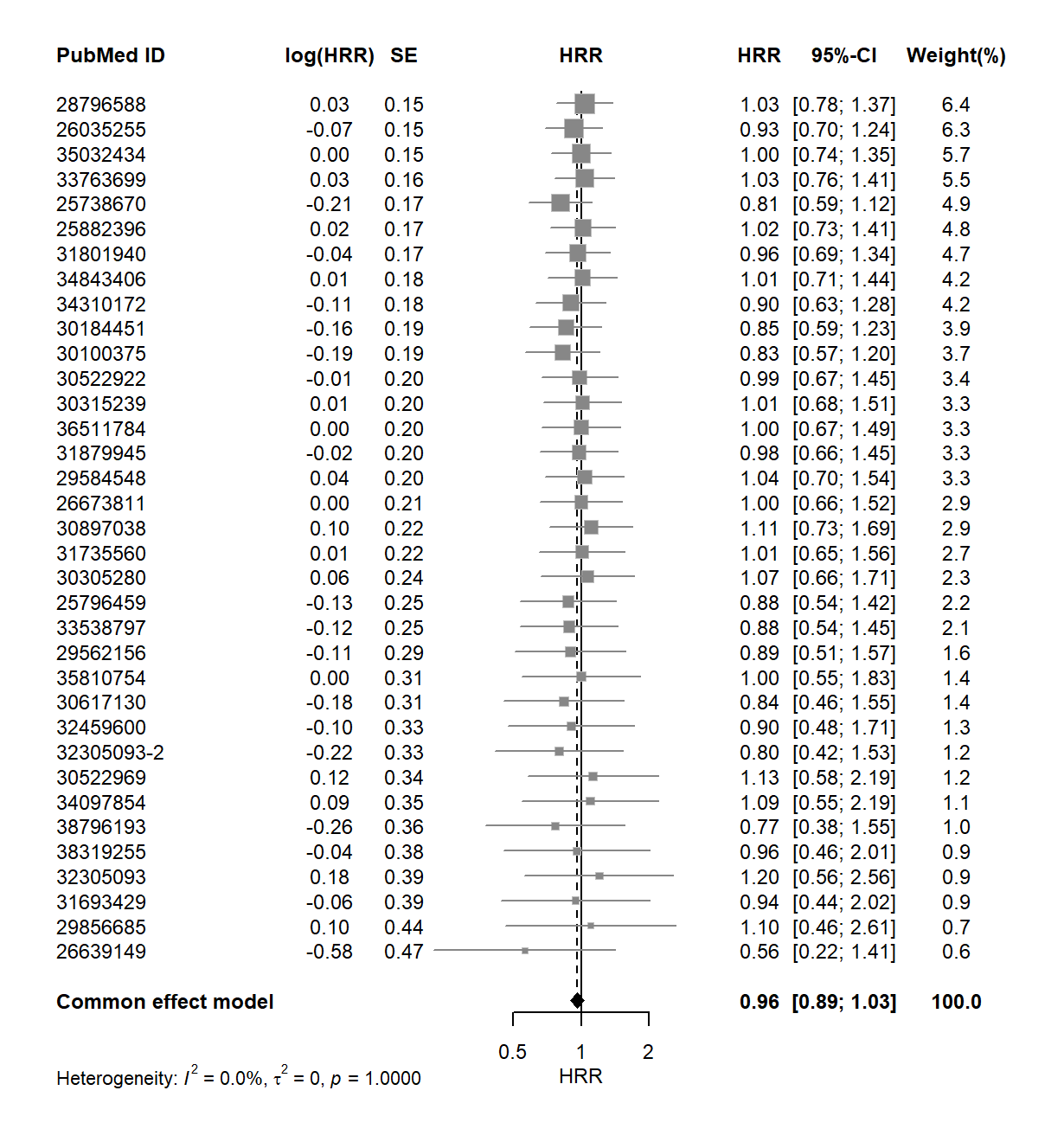


Figure S2. Forest plot of meta-analysis for HRR by masking.


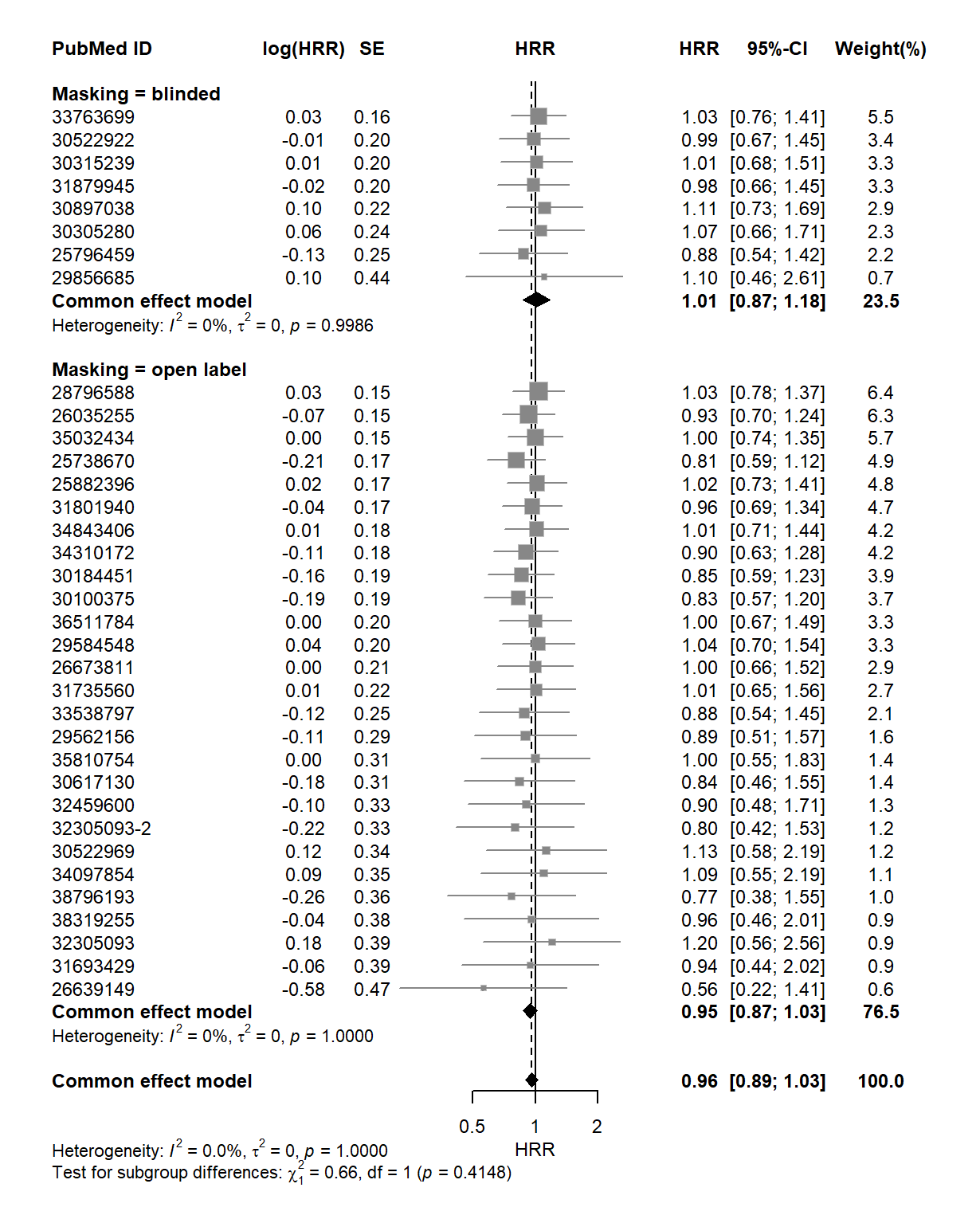


Figure S3. Forest plot of meta-analysis for HRR by cancer types.


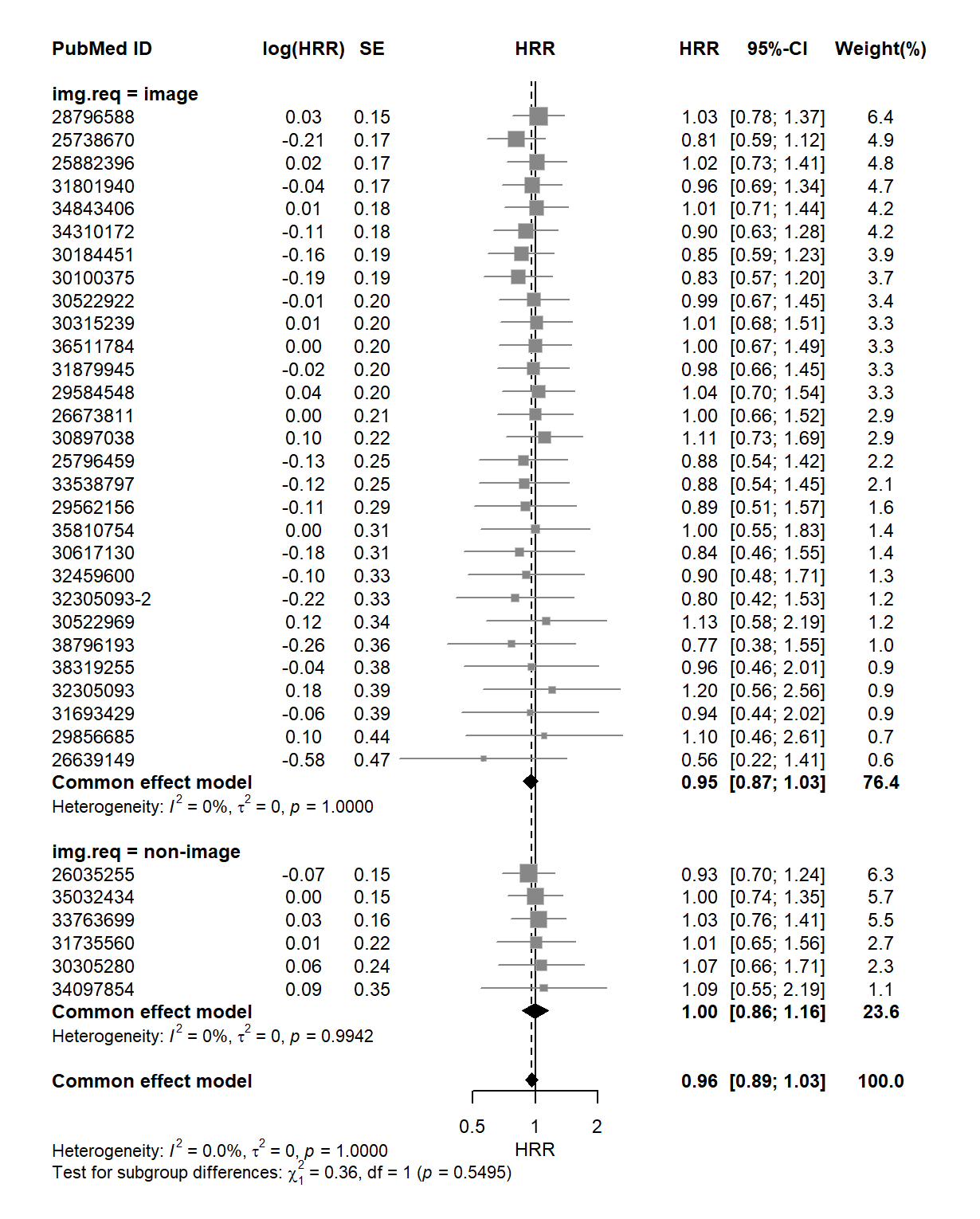


Figure S4. Forest plot of meta-analysis for HRR by sample size.


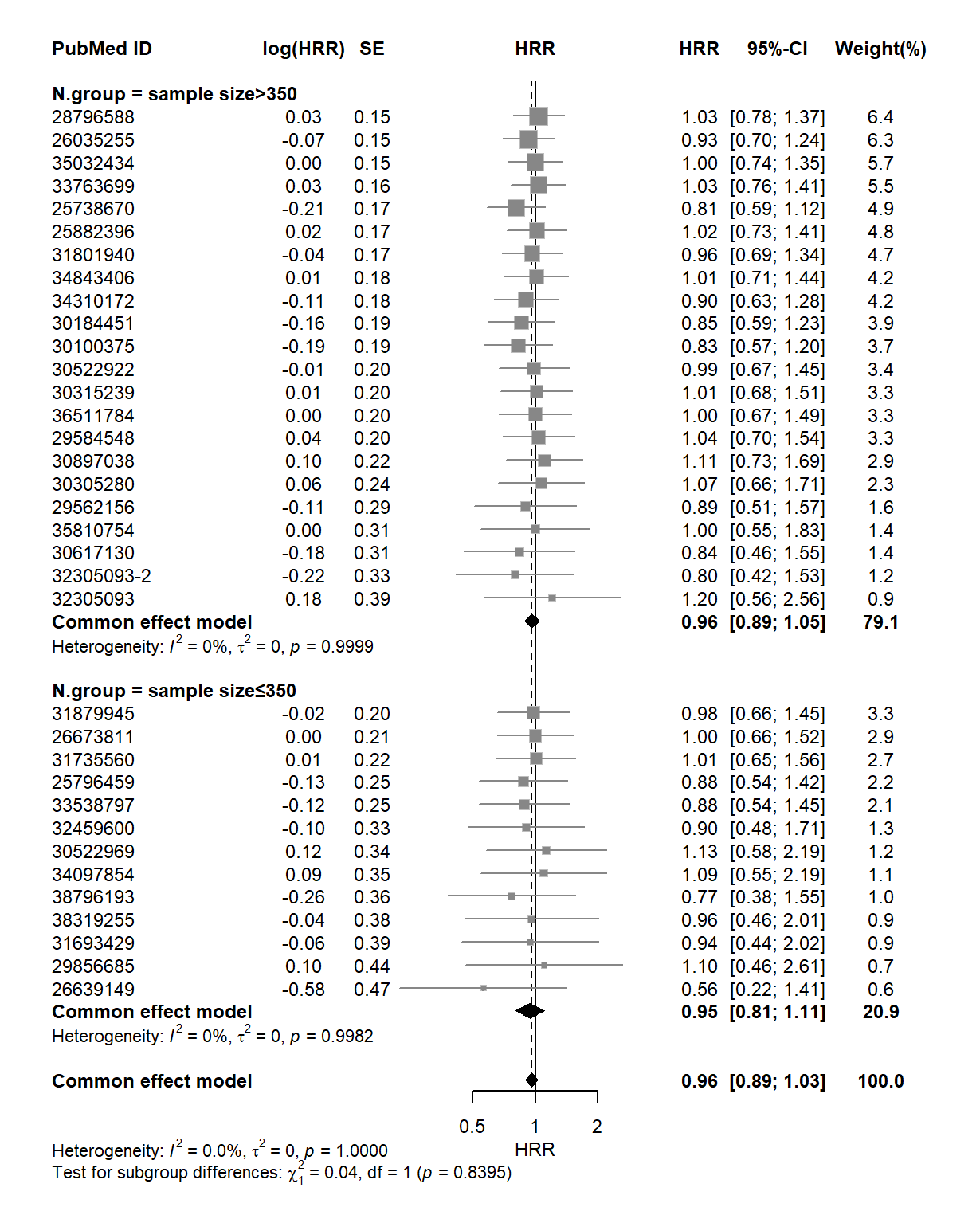


Figure S5. Forest plot of meta-analysis for $OR_{trt}$ in two-arm trials.


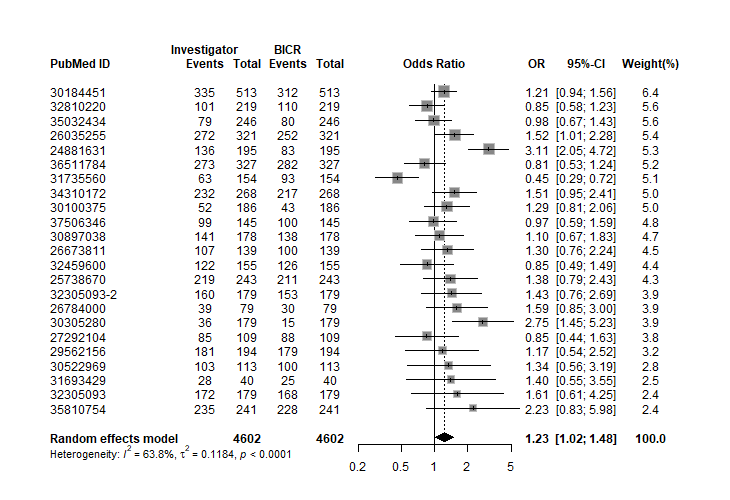


Figure S6. Forest plot of meta-analysis for $OR_{control}$ in two-arm trials.


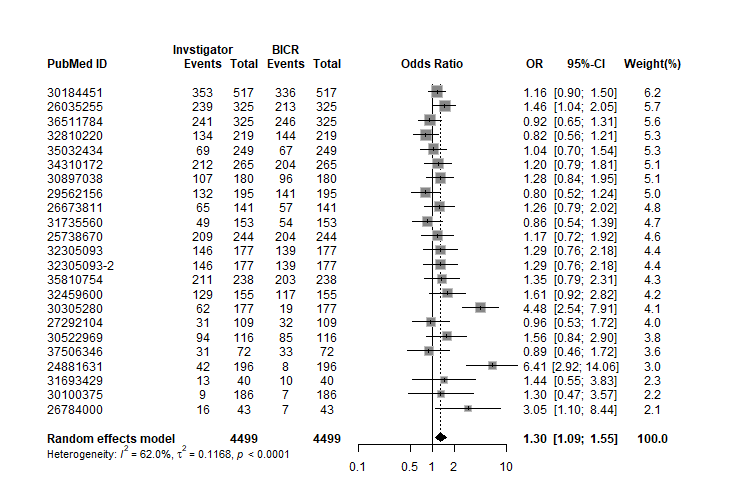


Figure S7. Forest plot of meta-analysis for OddsRR.


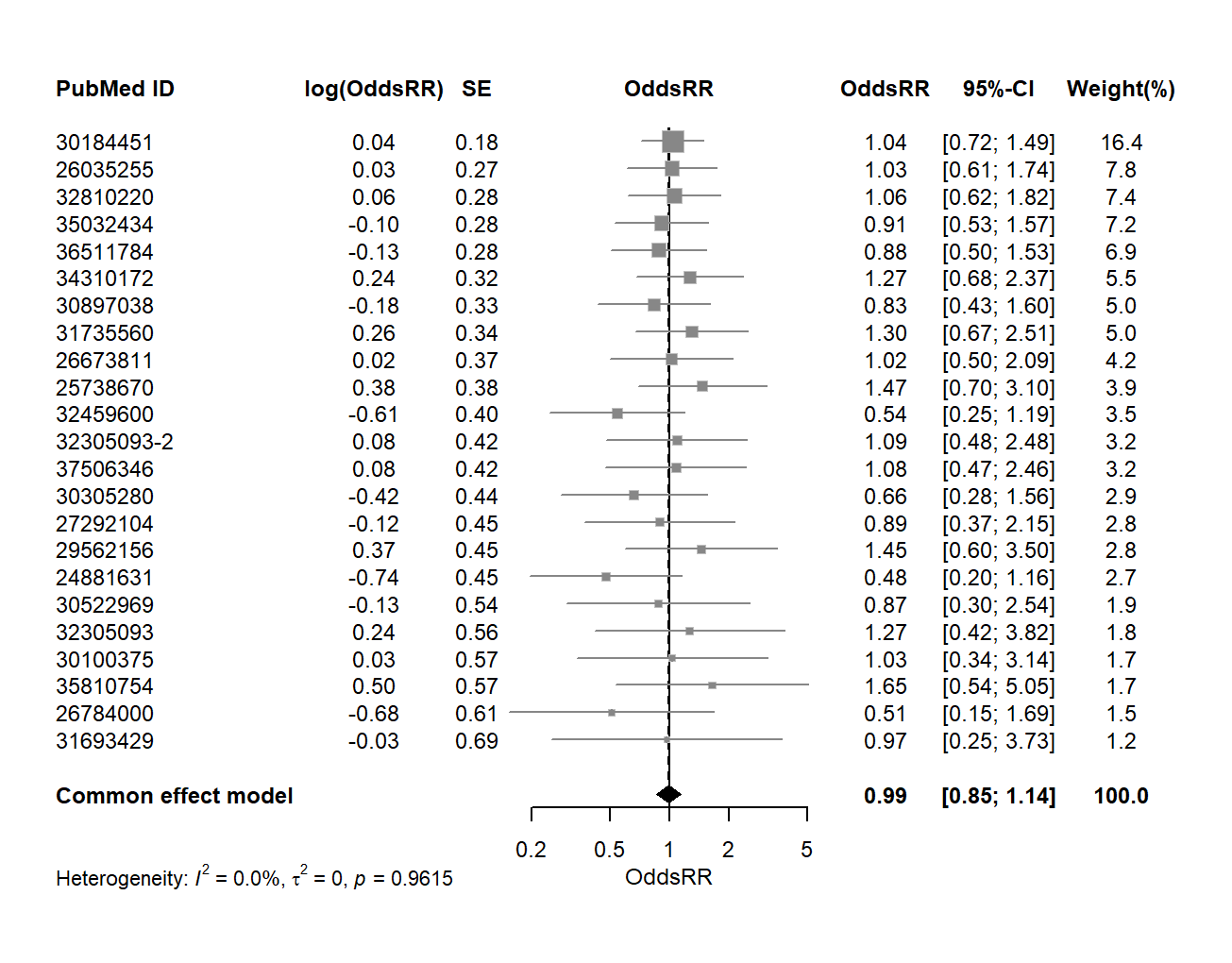


Figure S8. Forest plot of meta-analysis for OddsRR for open-label studies.


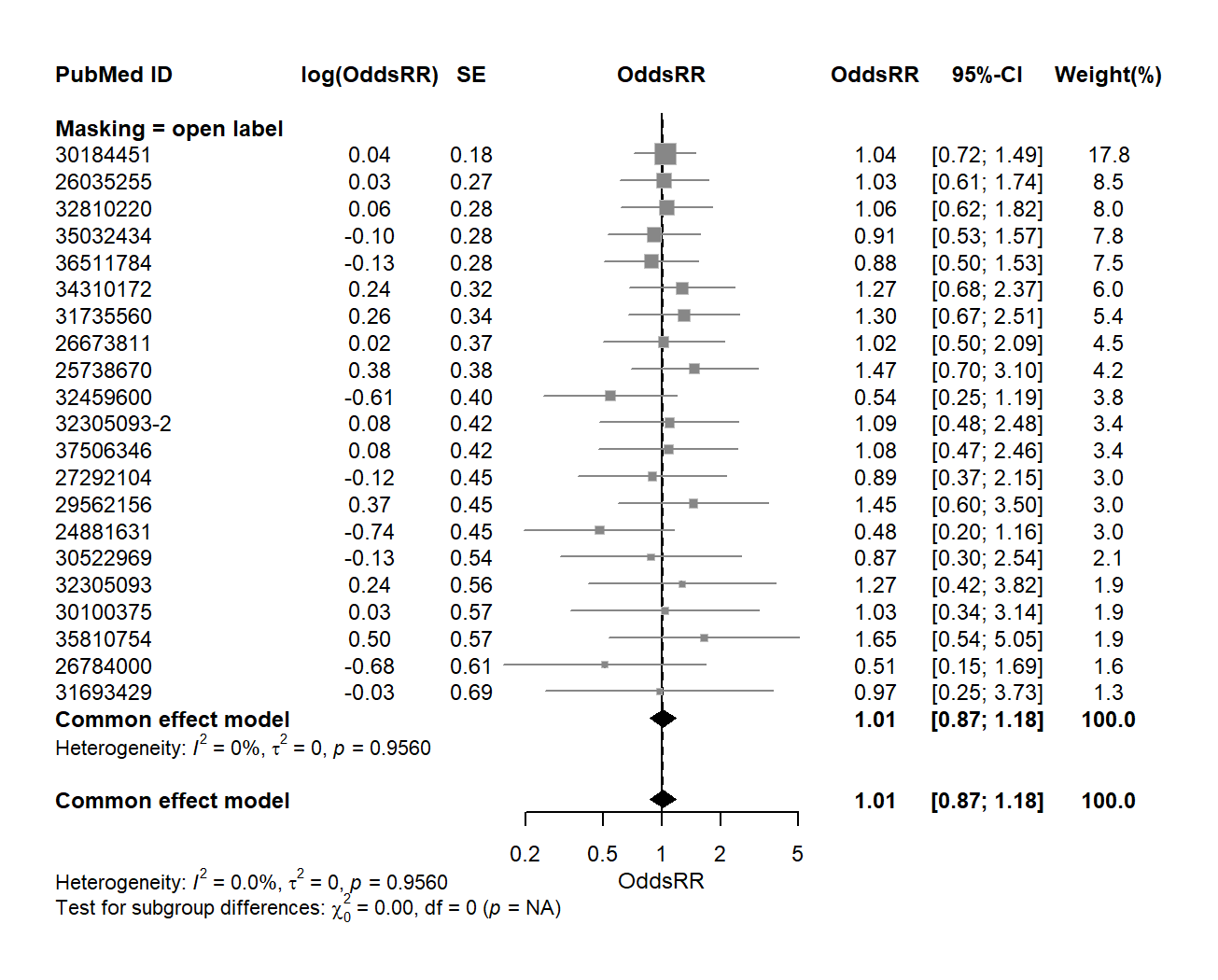


Figure S9. Forest plot of meta-analysis for OddsRR by cancer types.


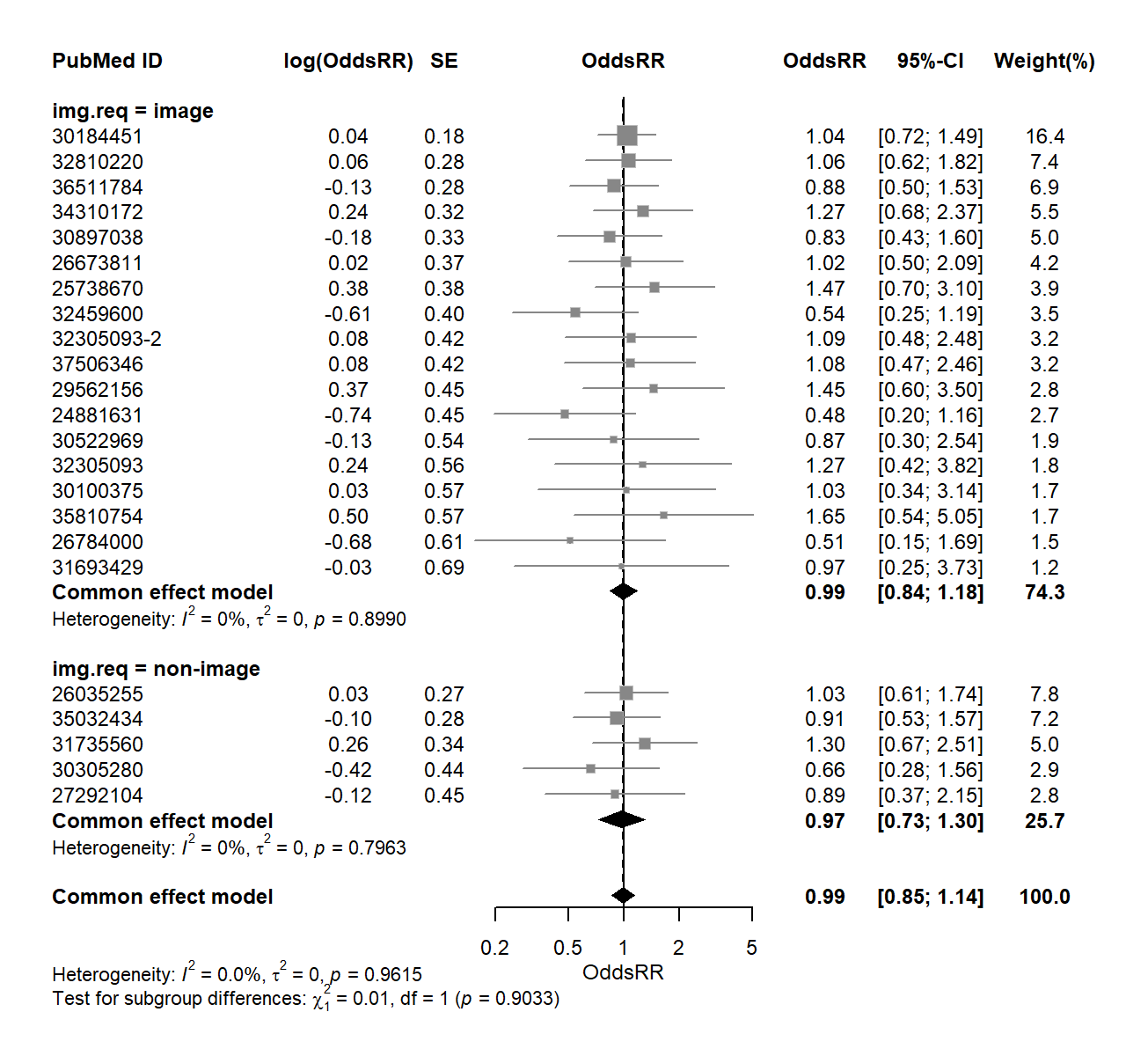


Figure S10. Forest plot of meta-analysis for OddsRR by sample size.


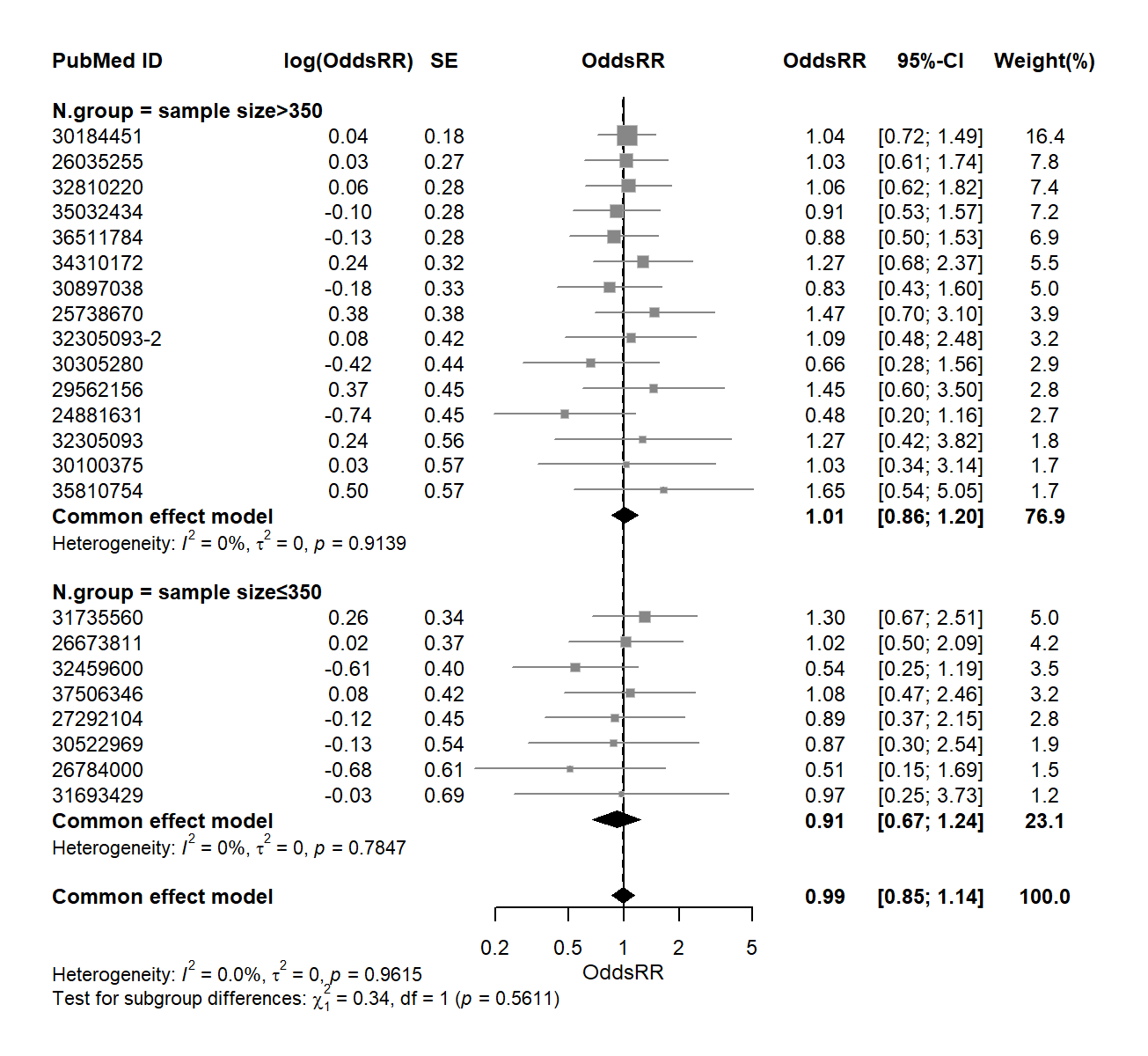


Figure S11. Forest plot of meta-analysis for $OR_{trt}$ in single-arm trials.


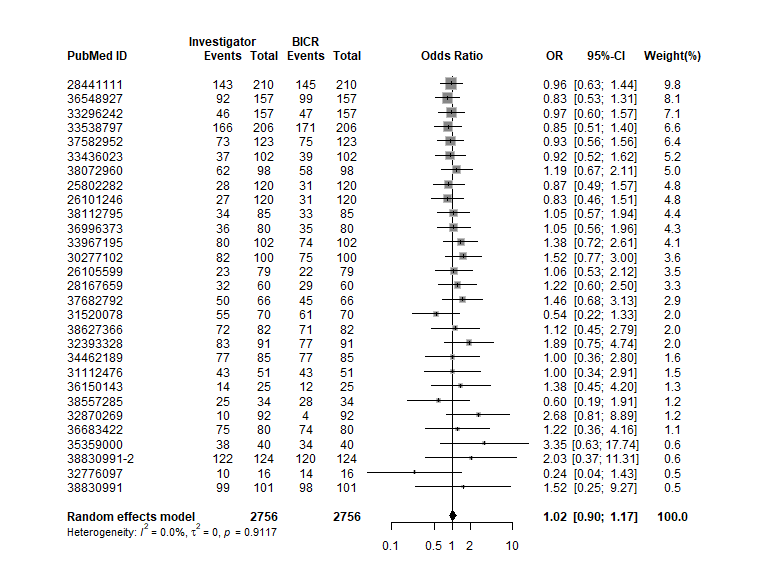


Figure S12. Risk of Bias for randomized trials.


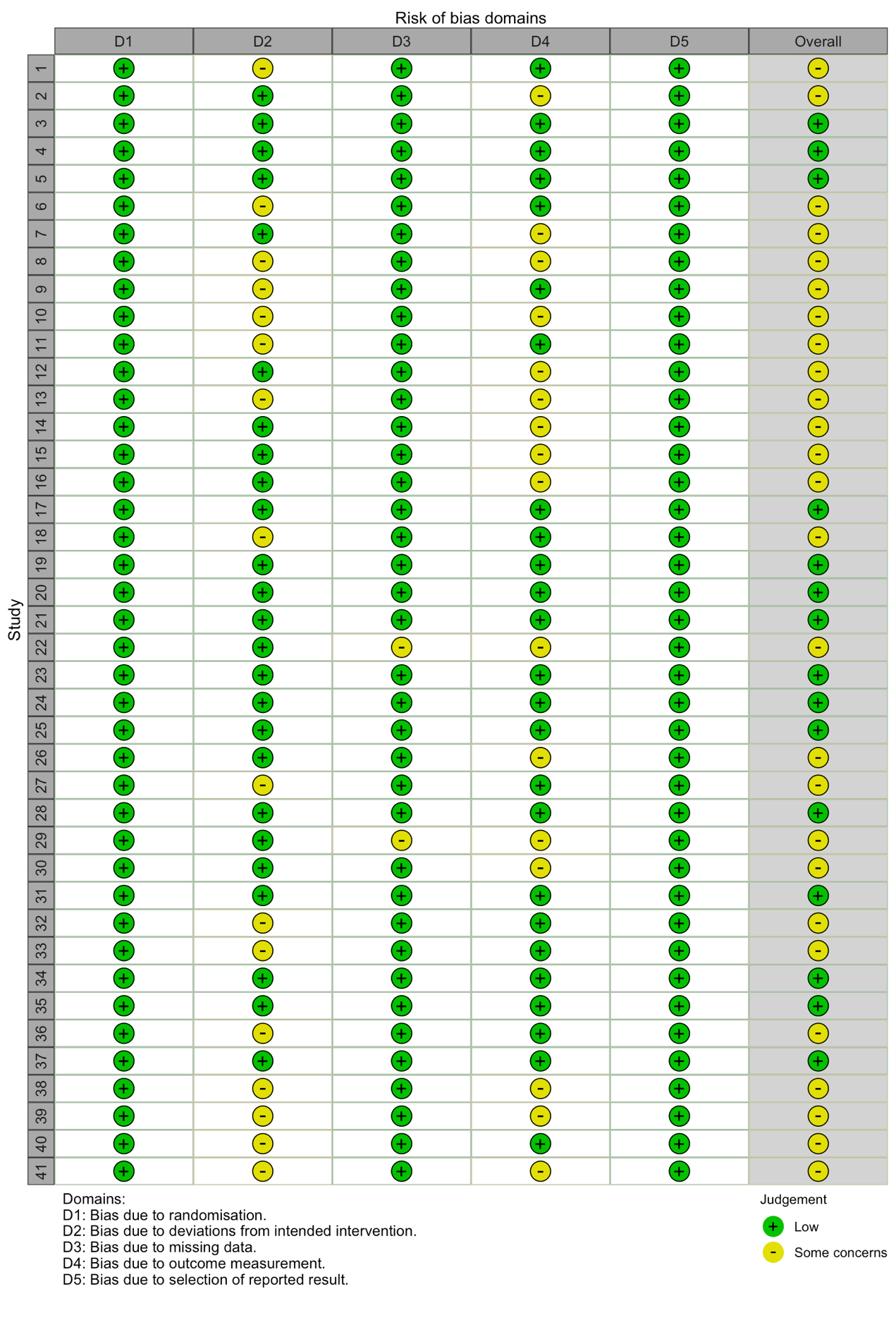

Supplement: oyaf375_Supplementary_Data [file oyaf375_supplementary_data.zip › supplementary figures.docx]
