# Supplementary material for: Concordance in assessments between investigators and blinded independent central review (BICR) in hematology oncology clinical trials: a meta-analysis
Source: Oncologist. 2025 Nov 9;30(11):oyaf375. doi: 10.1093/oncolo/oyaf375 (PMC12622372; doi:10.1093/oncolo/oyaf375)
Supplement: oyaf375_Supplementary_Data [file oyaf375_supplementary_data.zip › supplementary text and tables.docx]

**Text S1. Searching Equation.**

(((Lymphoma [Mesh] OR Multiple Myeloma[Mesh] OR Leukemia[Mesh] OR Lymphoma [tiab] OR Multiple Myeloma[tiab] OR Leukemia[tiab] OR Hematology [Mesh] OR Hematology[tiab]) AND (progression free survival [Mesh] OR disease progression[Mesh] OR progression free survival[tiab] OR PFS[tiab] OR objective response[tiab] OR ORR[tiab] OR investigator OR independent review) AND (“Phase 3” OR “Phase III”OR“Phase 2” OR “Phase II”)) AND (randomized controlled trial [pt] OR controlled clinical trial [pt] OR randomized [tiab] OR placebo [tiab] OR drug therapy [sh] OR randomly [tiab] OR trial [tiab]OR groups [tiab]))) AND ((“2014/01/01”[Date - Publication] : “2024/11/27”[Date - Publication]))

**Table S1. Characteristics of included studies.**

| **PubMed ID** | **First Author** | **Year** | **Primary Endpoint** | **Study Phase** | **Mask** | **Cancer Type** | **Specific Cancer Type** | **Sample Size** | **Therapy** | **Control** |
| --- | --- | --- | --- | --- | --- | --- | --- | --- | --- | --- |
| 24881631 | Byrd JC | 2014 | PFS by BICR | 3 | Open label | LEU/LYM | CLL/SLL | 391 | ibrutinib | ofatumumab |
| 25796459 | Moskowitz CH | 2015 | PFS by BICR | 3 | Double blinded | LYM | cHL | 329 | brentuximab vedotin | placebo |
| 25882396 | Hillmen P | 2015 | PFS by BICR | 3 | Open label | LEU | CLL | 447 | chlorambucil + ofatumumab | chlorambucil |
| 26035255 | Lonial S | 2015 | PFS/ORR by BICR | 3 | Open label | MM |  | 646 | elotuzumab + lenalidomide + dexamethasone | lenalidomide + dexamethasone |
| 25738670 | Robak T | 2015 | PFS by BICR | 3 | Open label | LYM | MCL | 487 | VR-CAP | R-CHOP |
| 26639149 | Burger JA | 2015 | PFS by BICR | 3 | Open label | LRU/LYM | CLL/SLL | 269 | ibrutinib | chlorambucil |
| 26673811 | Dreyling M | 2016 | PFS by BICR | 3 | Open label | LYM | MCL | 280 | ibrutinib | temsirolimus |
| 26784000 | Österborg A | 2016 | PFS by BICR | 3 | Open label | LEU | CLL | 122 | ofatumumab | physicians' choice |
| 27292104 | Kantarjian HM | 2016 | CRR by BICR/OS | 3 | Open label | LEU | ALL | 218 | inotuzumab ozogamicin | intensive chemotherapy |
| 28796588 | Vitolo U | 2017 | PFS by INV | 3 | Open label | LYM | DLBCL | 1418 | G-CHOP | R-CHOP |
| 30287523 | Flinn IW | 2018 | PFS by BICR | 3 | Open label | LEU/LYM | CLL/SLL | 319 | duvelisib | ofatumumab |
| 29584548 | Cheson BD | 2018 | PFS by BICR | 3 | Open label | LYM | iNHL | 413 | obinutuzumab + bendamustine | bendamustine |
| 30100375 | Kim YH | 2018 | PFS by INV | 3 | Open label | LYM | CTCL | 372 | mogamulizumab | vorinostat |
| 29562156 | Seymour JF | 2018 | PFS by INV | 3 | Open label | LEU | CLL | 389 | venetoclax + rituximab | bendamustine + rituximab |
| 30184451 | Morschhauser F | 2018 | CRR/PFS by BICR | 3 | Open label | LYM | FL | 1030 | rituximab + lenalidomide | rituximab + chemotherapy |
| 30305280 | Dickinson M | 2018 | Platelet Transfusion-Free rate by INV | 3 | Double blinded | MYE | MDS | 356 | eltrombopag + azacitidine | placebo + azacitidine |
| 29856685 | Dimopoulos MA | 2018 | PFS by BICR | 3 | Double blinded | LYM | WM | 150 | ibrutinib + rituximab | placebo + rituximab |
| 31240472 | Spicka I | 2019 | PFS by BICR | 3 | Open label | MM |  | 255 | plitidepsin + dexamethasone | dexamethasone |
| 30897038 | Leonard JP | 2019 | PFS by BICR | 3 | Quadruple blinded | LYM | FL/MZL | 358 | lenalidomide + rituximab | placebo + rituximab |
| 30617130 | Ramchandren R | 2019 | PFS by BICR | 3 | Open label | LYM | cHL | 497 | brentuximab vedotin + AVD | bleomycin + AVD |
| 30522922 | Horwitz S | 2019 | PFS by BICR | 3 | Double blinded | LYM | PTCL | 452 | brentuximab vedotin + CHP | CHOP |
| 30315239 | Fraser G | 2019 | PFS by BICR | 3 | Double blinded | LEU/LYM | CLL/SLL | 578 | ibrutinib+BR | placebo + BR |
| 31735560 | Attal M | 2019 | PFS by BICR | 3 | Open label | MM |  | 307 | isatuximab + pomalidomide + dexamethasone | pomalidomide + dexamethasone |
| 30522969 | Moreno C | 2019 | PFS by BICR | 3 | Open label | LEU | CLL | 229 | ibrutinib + obinutuzumab | chlorambucil + obinutuzumab |
| 31801940 | van Oers M | 2019 | PFS by INV | 3 | Open label | LEU | CLL | 480 | ofatumumab | observation |
| 32459600 | Ghia P | 2020 | PFS by BICR | 3 | Open label | LEU | CLL | 310 | acalabrutinib | idelalisib + rituximab or bendamustine + rituximab |
| 32305093 | Sharman JP | 2020 | PFS by BICR | 3 | Open label | LEU | CLL | 356 | acalabrutinib + obinutuzumab | obinutuzumab + chlorambucil |
| 32810220 | Maloney DG | 2020 | PFS by BICR | 3 | Open label | LYM | iNHL | 438 | ofatumumab | rituximab |
| 31879945 | Pettengell R | 2020 | PFS by BICR & INV | 3 | Single blinded | LYM | DLBCL/FL | 312 | pixantrone + rituximab | gemcitabine + rituximab |
| 34097854 | Moreau P | 2021 | PFS by BICR | 3 | Open label | MM |  | 302 | Isatuximab + carfilzomib + dexmethasone | carfilzomib + dexmethasone |
| 34843406 | Bachy E | 2021 | PFS by BICR | 3 | Open label | LYM | PTCL | 421 | Ro-CHOP | CHOP |
| 34310172 | Byrd JC | 2021 | PFS by BICR | 3 | Open label | LEU | CLL | 533 | acalabrutinib | ibrutinib |
| 33967195 | Shi YK | 2021 | ORR by BICR | 3 | Open label | LYM | iNHL | 102 | bendamustine hydrochloride |  |
| 33763699 | Facon T | 2021 | PFS by BICR | 3 | Double blinded | MM |  | 705 | ixazomib-Rd | placebo-Rd |
| 38319255 | Kater AP | 2022 | PFS by BICR | 3 | Open label | LEU | CLL | 211 | ibrutinib + venetoclax | chlorambucil + obinutuzumab |
| 35810754 | Tam CS | 2022 | PFS by BICR | 3 | Open label | LEU/LYM | CLL/SLL | 479 | zanubrutinib | bendamustine + rituximab |
| 35032434 | Schjesvold FH | 2022 | PFS by BICR | 3 | Open label | MM |  | 495 | melflufen + dexamethasone | pomalidomide + dexamethasone |
| 36511784 | Brown JR | 2023 | ORR by INV | 3 | Open label | LEU/LYM | CLL/SLL | 652 | zanubrutinib | ibrutinib |
| 37404773 | Townsend W | 2023 | PFS by INV | 3 | Open label | LYM | FL | 1202 | obinutuzumab + chemotherapy | rituximab + chemotherapy |
| 38112795 | Zhou H | 2023 | ORR by BICR | 3 | Open label | MM |  | 85 | generic pomalidomide + dexamethasone |  |
| 38796193 | Dupuis J | 2024 | PFS by INV | 3 | Open label | LYM | TFHL | 86 | azacitidine | investigators' choice |
| 26101246 | O’Connor OA | 2015 | ORR by BICR | 2 | Open label | LYM | PTCL | 120 | belinostat |  |
| 26105599 | Shi Y | 2015 | ORR by BICR | 2 | Open label | LYM | PTCL | 79 | chidamide |  |
| 25802282 | Lee HZ | 2015 | ORR by BICR | 2 | Open label | LYM | PTCL | 120 | belinostat |  |
| 28441111 | Chen R | 2017 | ORR by BICR | 2 | Open label | LYM | cHL | 210 | pembrolizumab |  |
| 28167659 | Noy A | 2017 | ORR by BICR | 2 | Open label | LYM | MZL | 60 | ibrutinib |  |
| 31112476 | Ramchandren R | 2019 | Safety / tolerability | 2 | Open label | LYM | cHL | 51 | nivolumab |  |
| 30277102 | Sharman JP | 2019 | ORR by INV | 2 | Open label | LYM | DLBCL | 100 | obinutuzumab + CHOP |  |
| 31693429 | Sehn LH | 2019 | CRR by BICR | 2 | Open label | LYM | DLBCL | 80 | polatuzumab vedotin + bendamustine + rituximab | bendamustine + rituximab |
| 33296242 | Richardson PG | 2020 | ORR by INV | 2 | Open label | MM |  | 157 | melflufen + dexamethasone |  |
| 32393328 | Xu W | 2020 | ORR by BICR | 2 | Open label | LEU/LYM | CLL/SLL | 91 | zanubrutinib |  |
| 31520078 | Song Y | 2020 | ORR by BICR | 2 | Open label | LYM | cHL | 70 | tislelizumab |  |
| 32776097 | Maruyama D | 2020 | ORR by BICR | 2 | Open label | LYM | cHL | 16 | nivolumab |  |
| 33538797 | Morschhauser F | 2021 | CR by BICR | 2 | Open label | LYM | DLBCL | 206 | venetoclax + R-CHOP |  |
| 32870269 | Armand P | 2021 | ORR by BICR | 2 | Open label | LYM | FL | 92 | nivolumab |  |
| 33436023 | Shi Y | 2021 | ORR by BICR | 2 | Open label | LYM | PTCL | 102 | geptanolimab |  |
| 34462189 | Lin N | 2022 | ORR by BICR | 2 | Open label | LYM | cHL | 85 | zimberelimab |  |
| 35359000 | Younes A | 2022 | CRR by BICR | 2 | Open label | LYM | FL | 40 | atezolizumab + obinutuzumab + bendamustine |  |
| 36548927 | Thieblemont C | 2022 | ORR by BICR | 2 | Open label | LYM | LBCL | 157 | epcoritamab |  |
| 36404384 | Fukuhara N | 2023 | CRR by BICR | 2 | Open label | LYM | FL | 8 | tisagenlecleucel |  |
| 36996373 | Huang H | 2023 | ORR by BICR | 2 | Open label | LYM | ENKTL | 80 | sugemalimab |  |
| 37582952 | Lesokhin AM | 2023 | ORR by BICR | 2 | Open label | MM |  | 123 | elranatamab |  |
| 37682792 | Opat S | 2023 | ORR by BICR | 2 | Open label | LYM | MZL | 66 | zanubrutinib |  |
| 37506346 | Zinzani PL | 2023 | ORR by BICR | 2 | Open label | LYM | FL | 217 | zanubrutinib + obinutuzumab | obinutuzumab |
| 36683422 | Xu W | 2023 | ORR by BICR | 2 | Open label | LEU/LYM | CLL/SLL | 80 | orelabrutinib |  |
| 36150143 | Izutsu K | 2023 | ORR by BICR | 2 | Open label | LEU/LYM | T-cell Leu/Lym | 25 | valemetostat |  |
| 38627366 | Wang H | 2024 | ORR by BICR | 2 | Open label | LYM | FL | 82 | TQ-B3525 |  |
| 38072960 | Budde LE | 2024 | ORR by BICR | 2 | Open label | LYM | LBCL | 98 | mosunetuzumab + polatuzumab vedotin |  |
| 38557285 | Song Y | 2024 | ORR by BICR | 2 | Open label | LYM | MCL | 34 | acalabrutinib |  |
| 38830991 | Morschhauser F | 2024 | ORR by BICR | 2 | Open label | LYM | FL | 101 | lisocabtagene maraleucel |  |

PFS, progression-free survival; ORR: objective response rate; CRR: complete response rate; OS: overall survival; LYM, Lymphoma; LEU, Leukemia; CLL, Chronic Lymphocytic Leukemia; SLL, Small Lymphocytic Lymphoma; cHL, Classical Hodgkin Lymphoma; MM, Multiple Myeloma; MCL, Mantle Cell Lymphoma; ALL, Acute Lymphoblastic Leukemia; iNHL, Indolent Non-Hodgkin Lymphoma; CTCL, Cutaneous T-Cell Lymphoma; FL, Follicular Lymphoma; MDS, Myelodysplastic Syndromes; WM, Waldenström Macroglobulinemia; MZL, Marginal Zone Lymphoma; PTCL, Peripheral T-Cell Lymphoma; DLBCL, Diffuse Large B-Cell Lymphoma; ENKTL, Extranodal Natural Killer/T-Cell Lymphoma; T-cell Leu/Lym, T-Cell Leukemia/Lymphoma; LBCL, Large B-Cell Lymphoma; MYE, Myeloid; TFHL, Follicular helper T-cell lymphoma

**Table S2. Consistency of statistical significance for HR by BICR and Investigators (INV).**

| **n=33** | **Statistically significant (BICR+)** | **Not statistically significant (BICR－)** |
| --- | --- | --- |
| **Statistically significant (INV+)** | 26 | 0 |
| **Not statistically significant (INV－)** | 0 | 7 |

n: number of studies.

**Table S3. Agreement assessment of ORR between BICR and INV in single arms.**

| **Group** | **Number of studies(n)** | **OR (95% CI)** |
| --- | --- | --- |
| **Treatment** |  |  |
| two-arm trials | 23 | 1.23 (1.02, 1.48) |
| single-arm trials | 29 | 1.02 (0.90, 1.17) |
| **Control (two-arm trials)** | 23 | 1.30 (1.09, 1.55) |

**Table S4. Risk of Bias Analysis for Randomized Trials.**

| **PubMed ID** | 24881631 | | **First author** | Byrd JC | |
| --- | --- | --- | --- | --- | --- |
| **Domain** | | **Assessment items** | | | **Risk of bias judgement** |
| **Bias arising from the randomization process** | | Was the randomization process appropriate, including generation and concealment of the allocation sequence, with no evidence of baseline imbalance? | | | Low |
| **Bias due to deviations from intended interventions** | | Were participants and personnel blinded, and were any deviations from intended interventions likely to affect outcomes or be unbalanced between groups, with appropriate analysis of assigned groups? | | | Some concerns |
| **Bias due to missing outcome data** | | Was outcome data available for all, or nearly all, participants randomized, and if not, is it unlikely that missingness depended on the true outcome or biased the results? | | | Low |
| **Bias in measurement of the outcome** | | Was the outcome measured appropriately, consistently across groups, and without likely influence from assessors’ knowledge of intervention assignment? | | | Low |
| **Bias in selection of the reported result** | | Was the result analyzed following a pre-specified analysis plan, without selective reporting from multiple eligible outcome measurements or analyses? | | | Low |
| **Overall bias** | |  | | | Some concerns |
| **PubMed ID** | 25796459 | | **First author** | Moskowitz CH | |
| **Domain** | | **Assessment items** | | | **Risk of bias judgement** |
| **Bias arising from the randomization process** | | Was the randomization process appropriate, including generation and concealment of the allocation sequence, with no evidence of baseline imbalance? | | | Low |
| **Bias due to deviations from intended interventions** | | Were participants and personnel blinded, and were any deviations from intended interventions likely to affect outcomes or be unbalanced between groups, with appropriate analysis of assigned groups? | | | Low |
| **Bias due to missing outcome data** | | Was outcome data available for all, or nearly all, participants randomized, and if not, is it unlikely that missingness depended on the true outcome or biased the results? | | | Low |
| **Bias in measurement of the outcome** | | Was the outcome measured appropriately, consistently across groups, and without likely influence from assessors’ knowledge of intervention assignment? | | | Some concerns |
| **Bias in selection of the reported result** | | Was the result analyzed following a pre-specified analysis plan, without selective reporting from multiple eligible outcome measurements or analyses? | | | Low |
| **Overall bias** | |  | | | Some concerns |
| **PubMed ID** | 25882396 | | **First author** | Hillmen P | |
| **Domain** | | **Assessment items** | | | **Risk of bias judgement** |
| **Bias arising from the randomization process** | | Was the randomization process appropriate, including generation and concealment of the allocation sequence, with no evidence of baseline imbalance? | | | Low |
| **Bias due to deviations from intended interventions** | | Were participants and personnel blinded, and were any deviations from intended interventions likely to affect outcomes or be unbalanced between groups, with appropriate analysis of assigned groups? | | | Low |
| **Bias due to missing outcome data** | | Was outcome data available for all, or nearly all, participants randomized, and if not, is it unlikely that missingness depended on the true outcome or biased the results? | | | Low |
| **Bias in measurement of the outcome** | | Was the outcome measured appropriately, consistently across groups, and without likely influence from assessors’ knowledge of intervention assignment? | | | Low |
| **Bias in selection of the reported result** | | Was the result analyzed following a pre-specified analysis plan, without selective reporting from multiple eligible outcome measurements or analyses? | | | Low |
| **Overall bias** | |  | | | Low |
| **PubMed ID** | 26035255 | | **First author** | Lonial S | |
| **Domain** | | **Assessment items** | | | **Risk of bias judgement** |
| **Bias arising from the randomization process** | | Was the randomization process appropriate, including generation and concealment of the allocation sequence, with no evidence of baseline imbalance? | | | Low |
| **Bias due to deviations from intended interventions** | | Were participants and personnel blinded, and were any deviations from intended interventions likely to affect outcomes or be unbalanced between groups, with appropriate analysis of assigned groups? | | | Low |
| **Bias due to missing outcome data** | | Was outcome data available for all, or nearly all, participants randomized, and if not, is it unlikely that missingness depended on the true outcome or biased the results? | | | Low |
| **Bias in measurement of the outcome** | | Was the outcome measured appropriately, consistently across groups, and without likely influence from assessors’ knowledge of intervention assignment? | | | Low |
| **Bias in selection of the reported result** | | Was the result analyzed following a pre-specified analysis plan, without selective reporting from multiple eligible outcome measurements or analyses? | | | Low |
| **Overall bias** | |  | | | Low |
| **PubMed ID** | 25738670 | | **First author** | Robak T | |
| **Domain** | | **Assessment items** | | | **Risk of bias judgement** |
| **Bias arising from the randomization process** | | Was the randomization process appropriate, including generation and concealment of the allocation sequence, with no evidence of baseline imbalance? | | | Low |
| **Bias due to deviations from intended interventions** | | Were participants and personnel blinded, and were any deviations from intended interventions likely to affect outcomes or be unbalanced between groups, with appropriate analysis of assigned groups? | | | Low |
| **Bias due to missing outcome data** | | Was outcome data available for all, or nearly all, participants randomized, and if not, is it unlikely that missingness depended on the true outcome or biased the results? | | | Low |
| **Bias in measurement of the outcome** | | Was the outcome measured appropriately, consistently across groups, and without likely influence from assessors’ knowledge of intervention assignment? | | | Low |
| **Bias in selection of the reported result** | | Was the result analyzed following a pre-specified analysis plan, without selective reporting from multiple eligible outcome measurements or analyses? | | | Low |
| **Overall bias** | |  | | | Low |
| **PubMed ID** | 26639149 | | **First author** | Burger JA | |
| **Domain** | | **Assessment items** | | | **Risk of bias judgement** |
| **Bias arising from the randomization process** | | Was the randomization process appropriate, including generation and concealment of the allocation sequence, with no evidence of baseline imbalance? | | | Low |
| **Bias due to deviations from intended interventions** | | Were participants and personnel blinded, and were any deviations from intended interventions likely to affect outcomes or be unbalanced between groups, with appropriate analysis of assigned groups? | | | Some concerns |
| **Bias due to missing outcome data** | | Was outcome data available for all, or nearly all, participants randomized, and if not, is it unlikely that missingness depended on the true outcome or biased the results? | | | Low |
| **Bias in measurement of the outcome** | | Was the outcome measured appropriately, consistently across groups, and without likely influence from assessors’ knowledge of intervention assignment? | | | Low |
| **Bias in selection of the reported result** | | Was the result analyzed following a pre-specified analysis plan, without selective reporting from multiple eligible outcome measurements or analyses? | | | Low |
| **Overall bias** | |  | | | Some concerns |
| **PubMed ID** | 26673811 | | **First author** | Dreyling M | |
| **Domain** | | **Assessment items** | | | **Risk of bias judgement** |
| **Bias arising from the randomization process** | | Was the randomization process appropriate, including generation and concealment of the allocation sequence, with no evidence of baseline imbalance? | | | Low |
| **Bias due to deviations from intended interventions** | | Were participants and personnel blinded, and were any deviations from intended interventions likely to affect outcomes or be unbalanced between groups, with appropriate analysis of assigned groups? | | | Low |
| **Bias due to missing outcome data** | | Was outcome data available for all, or nearly all, participants randomized, and if not, is it unlikely that missingness depended on the true outcome or biased the results? | | | Low |
| **Bias in measurement of the outcome** | | Was the outcome measured appropriately, consistently across groups, and without likely influence from assessors’ knowledge of intervention assignment? | | | Some concerns |
| **Bias in selection of the reported result** | | Was the result analyzed following a pre-specified analysis plan, without selective reporting from multiple eligible outcome measurements or analyses? | | | Low |
| **Overall bias** | |  | | | Some concerns |
| **PubMed ID** | 26784000 | | **First author** | Österborg A | |
| **Domain** | | **Assessment items** | | | **Risk of bias judgement** |
| **Bias arising from the randomization process** | | Was the randomization process appropriate, including generation and concealment of the allocation sequence, with no evidence of baseline imbalance? | | | Low |
| **Bias due to deviations from intended interventions** | | Were participants and personnel blinded, and were any deviations from intended interventions likely to affect outcomes or be unbalanced between groups, with appropriate analysis of assigned groups? | | | Some concerns |
| **Bias due to missing outcome data** | | Was outcome data available for all, or nearly all, participants randomized, and if not, is it unlikely that missingness depended on the true outcome or biased the results? | | | Low |
| **Bias in measurement of the outcome** | | Was the outcome measured appropriately, consistently across groups, and without likely influence from assessors’ knowledge of intervention assignment? | | | Some concerns |
| **Bias in selection of the reported result** | | Was the result analyzed following a pre-specified analysis plan, without selective reporting from multiple eligible outcome measurements or analyses? | | | Low |
| **Overall bias** | |  | | | Some concerns |
| **PubMed ID** | 27292104 | | **First author** | Kantarjian HM | |
| **Domain** | | **Assessment items** | | | **Risk of bias judgement** |
| **Bias arising from the randomization process** | | Was the randomization process appropriate, including generation and concealment of the allocation sequence, with no evidence of baseline imbalance? | | | Low |
| **Bias due to deviations from intended interventions** | | Were participants and personnel blinded, and were any deviations from intended interventions likely to affect outcomes or be unbalanced between groups, with appropriate analysis of assigned groups? | | | Some concerns |
| **Bias due to missing outcome data** | | Was outcome data available for all, or nearly all, participants randomized, and if not, is it unlikely that missingness depended on the true outcome or biased the results? | | | Low |
| **Bias in measurement of the outcome** | | Was the outcome measured appropriately, consistently across groups, and without likely influence from assessors’ knowledge of intervention assignment? | | | Low |
| **Bias in selection of the reported result** | | Was the result analyzed following a pre-specified analysis plan, without selective reporting from multiple eligible outcome measurements or analyses? | | | Low |
| **Overall bias** | |  | | | Some concerns |
| **PubMed ID** | 28796588 | | **First author** | Vitolo U | |
| **Domain** | | **Assessment items** | | | **Risk of bias judgement** |
| **Bias arising from the randomization process** | | Was the randomization process appropriate, including generation and concealment of the allocation sequence, with no evidence of baseline imbalance? | | | Low |
| **Bias due to deviations from intended interventions** | | Were participants and personnel blinded, and were any deviations from intended interventions likely to affect outcomes or be unbalanced between groups, with appropriate analysis of assigned groups? | | | Some concerns |
| **Bias due to missing outcome data** | | Was outcome data available for all, or nearly all, participants randomized, and if not, is it unlikely that missingness depended on the true outcome or biased the results? | | | Low |
| **Bias in measurement of the outcome** | | Was the outcome measured appropriately, consistently across groups, and without likely influence from assessors’ knowledge of intervention assignment? | | | Some concerns |
| **Bias in selection of the reported result** | | Was the result analyzed following a pre-specified analysis plan, without selective reporting from multiple eligible outcome measurements or analyses? | | | Low |
| **Overall bias** | |  | | | Some concerns |
| **PubMed ID** | 30287523 | | **First author** | Flinn IW | |
| **Domain** | | **Assessment items** | | | **Risk of bias judgement** |
| **Bias arising from the randomization process** | | Was the randomization process appropriate, including generation and concealment of the allocation sequence, with no evidence of baseline imbalance? | | | Low |
| **Bias due to deviations from intended interventions** | | Were participants and personnel blinded, and were any deviations from intended interventions likely to affect outcomes or be unbalanced between groups, with appropriate analysis of assigned groups? | | | Some concerns |
| **Bias due to missing outcome data** | | Was outcome data available for all, or nearly all, participants randomized, and if not, is it unlikely that missingness depended on the true outcome or biased the results? | | | Low |
| **Bias in measurement of the outcome** | | Was the outcome measured appropriately, consistently across groups, and without likely influence from assessors’ knowledge of intervention assignment? | | | Low |
| **Bias in selection of the reported result** | | Was the result analyzed following a pre-specified analysis plan, without selective reporting from multiple eligible outcome measurements or analyses? | | | Low |
| **Overall bias** | |  | | | Some concerns |
| **PubMed ID** | 29584548 | | **First author** | Cheson BD | |
| **Domain** | | **Assessment items** | | | **Risk of bias judgement** |
| **Bias arising from the randomization process** | | Was the randomization process appropriate, including generation and concealment of the allocation sequence, with no evidence of baseline imbalance? | | | Low |
| **Bias due to deviations from intended interventions** | | Were participants and personnel blinded, and were any deviations from intended interventions likely to affect outcomes or be unbalanced between groups, with appropriate analysis of assigned groups? | | | Low |
| **Bias due to missing outcome data** | | Was outcome data available for all, or nearly all, participants randomized, and if not, is it unlikely that missingness depended on the true outcome or biased the results? | | | Low |
| **Bias in measurement of the outcome** | | Was the outcome measured appropriately, consistently across groups, and without likely influence from assessors’ knowledge of intervention assignment? | | | Some concerns |
| **Bias in selection of the reported result** | | Was the result analyzed following a pre-specified analysis plan, without selective reporting from multiple eligible outcome measurements or analyses? | | | Low |
| **Overall bias** | |  | | | Some concerns |
| **PubMed ID** | 30100375 | | **First author** | Kim YH | |
| **Domain** | | **Assessment items** | | | **Risk of bias judgement** |
| **Bias arising from the randomization process** | | Was the randomization process appropriate, including generation and concealment of the allocation sequence, with no evidence of baseline imbalance? | | | Low |
| **Bias due to deviations from intended interventions** | | Were participants and personnel blinded, and were any deviations from intended interventions likely to affect outcomes or be unbalanced between groups, with appropriate analysis of assigned groups? | | | Some concerns |
| **Bias due to missing outcome data** | | Was outcome data available for all, or nearly all, participants randomized, and if not, is it unlikely that missingness depended on the true outcome or biased the results? | | | Low |
| **Bias in measurement of the outcome** | | Was the outcome measured appropriately, consistently across groups, and without likely influence from assessors’ knowledge of intervention assignment? | | | Some concerns |
| **Bias in selection of the reported result** | | Was the result analyzed following a pre-specified analysis plan, without selective reporting from multiple eligible outcome measurements or analyses? | | | Low |
| **Overall bias** | |  | | | Some concerns |
| **PubMed ID** | 29562156 | | **First author** | Seymour JF | |
| **Domain** | | **Assessment items** | | | **Risk of bias judgement** |
| **Bias arising from the randomization process** | | Was the randomization process appropriate, including generation and concealment of the allocation sequence, with no evidence of baseline imbalance? | | | Low |
| **Bias due to deviations from intended interventions** | | Were participants and personnel blinded, and were any deviations from intended interventions likely to affect outcomes or be unbalanced between groups, with appropriate analysis of assigned groups? | | | Low |
| **Bias due to missing outcome data** | | Was outcome data available for all, or nearly all, participants randomized, and if not, is it unlikely that missingness depended on the true outcome or biased the results? | | | Low |
| **Bias in measurement of the outcome** | | Was the outcome measured appropriately, consistently across groups, and without likely influence from assessors’ knowledge of intervention assignment? | | | Some concerns |
| **Bias in selection of the reported result** | | Was the result analyzed following a pre-specified analysis plan, without selective reporting from multiple eligible outcome measurements or analyses? | | | Low |
| **Overall bias** | |  | | | Some concerns |
| **PubMed ID** | 30184451 | | **First author** | Morschhauser F | |
| **Domain** | | **Assessment items** | | | **Risk of bias judgement** |
| **Bias arising from the randomization process** | | Was the randomization process appropriate, including generation and concealment of the allocation sequence, with no evidence of baseline imbalance? | | | Low |
| **Bias due to deviations from intended interventions** | | Were participants and personnel blinded, and were any deviations from intended interventions likely to affect outcomes or be unbalanced between groups, with appropriate analysis of assigned groups? | | | Low |
| **Bias due to missing outcome data** | | Was outcome data available for all, or nearly all, participants randomized, and if not, is it unlikely that missingness depended on the true outcome or biased the results? | | | Low |
| **Bias in measurement of the outcome** | | Was the outcome measured appropriately, consistently across groups, and without likely influence from assessors’ knowledge of intervention assignment? | | | Some concerns |
| **Bias in selection of the reported result** | | Was the result analyzed following a pre-specified analysis plan, without selective reporting from multiple eligible outcome measurements or analyses? | | | Low |
| **Overall bias** | |  | | | Some concerns |
| **PubMed ID** | 30305280 | | **First author** | Dickinson M | |
| **Domain** | | **Assessment items** | | | **Risk of bias judgement** |
| **Bias arising from the randomization process** | | Was the randomization process appropriate, including generation and concealment of the allocation sequence, with no evidence of baseline imbalance? | | | Low |
| **Bias due to deviations from intended interventions** | | Were participants and personnel blinded, and were any deviations from intended interventions likely to affect outcomes or be unbalanced between groups, with appropriate analysis of assigned groups? | | | Low |
| **Bias due to missing outcome data** | | Was outcome data available for all, or nearly all, participants randomized, and if not, is it unlikely that missingness depended on the true outcome or biased the results? | | | Low |
| **Bias in measurement of the outcome** | | Was the outcome measured appropriately, consistently across groups, and without likely influence from assessors’ knowledge of intervention assignment? | | | Some concerns |
| **Bias in selection of the reported result** | | Was the result analyzed following a pre-specified analysis plan, without selective reporting from multiple eligible outcome measurements or analyses? | | | Low |
| **Overall bias** | |  | | | Some concerns |
| **PubMed ID** | 29856685 | | **First author** | Dimopoulos MA | |
| **Domain** | | **Assessment items** | | | **Risk of bias judgement** |
| **Bias arising from the randomization process** | | Was the randomization process appropriate, including generation and concealment of the allocation sequence, with no evidence of baseline imbalance? | | | Low |
| **Bias due to deviations from intended interventions** | | Were participants and personnel blinded, and were any deviations from intended interventions likely to affect outcomes or be unbalanced between groups, with appropriate analysis of assigned groups? | | | Low |
| **Bias due to missing outcome data** | | Was outcome data available for all, or nearly all, participants randomized, and if not, is it unlikely that missingness depended on the true outcome or biased the results? | | | Low |
| **Bias in measurement of the outcome** | | Was the outcome measured appropriately, consistently across groups, and without likely influence from assessors’ knowledge of intervention assignment? | | | Low |
| **Bias in selection of the reported result** | | Was the result analyzed following a pre-specified analysis plan, without selective reporting from multiple eligible outcome measurements or analyses? | | | Low |
| **Overall bias** | |  | | | Low |
| **PubMed ID** | 31240472 | | **First author** | Spicka I | |
| **Domain** | | **Assessment items** | | | **Risk of bias judgement** |
| **Bias arising from the randomization process** | | Was the randomization process appropriate, including generation and concealment of the allocation sequence, with no evidence of baseline imbalance? | | | Low |
| **Bias due to deviations from intended interventions** | | Were participants and personnel blinded, and were any deviations from intended interventions likely to affect outcomes or be unbalanced between groups, with appropriate analysis of assigned groups? | | | Some concerns |
| **Bias due to missing outcome data** | | Was outcome data available for all, or nearly all, participants randomized, and if not, is it unlikely that missingness depended on the true outcome or biased the results? | | | Low |
| **Bias in measurement of the outcome** | | Was the outcome measured appropriately, consistently across groups, and without likely influence from assessors’ knowledge of intervention assignment? | | | Low |
| **Bias in selection of the reported result** | | Was the result analyzed following a pre-specified analysis plan, without selective reporting from multiple eligible outcome measurements or analyses? | | | Low |
| **Overall bias** | |  | | | Some concerns |
| **PubMed ID** | 30897038 | | **First author** | Leonard JP | |
| **Domain** | | **Assessment items** | | | **Risk of bias judgement** |
| **Bias arising from the randomization process** | | Was the randomization process appropriate, including generation and concealment of the allocation sequence, with no evidence of baseline imbalance? | | | Low |
| **Bias due to deviations from intended interventions** | | Were participants and personnel blinded, and were any deviations from intended interventions likely to affect outcomes or be unbalanced between groups, with appropriate analysis of assigned groups? | | | Low |
| **Bias due to missing outcome data** | | Was outcome data available for all, or nearly all, participants randomized, and if not, is it unlikely that missingness depended on the true outcome or biased the results? | | | Low |
| **Bias in measurement of the outcome** | | Was the outcome measured appropriately, consistently across groups, and without likely influence from assessors’ knowledge of intervention assignment? | | | Low |
| **Bias in selection of the reported result** | | Was the result analyzed following a pre-specified analysis plan, without selective reporting from multiple eligible outcome measurements or analyses? | | | Low |
| **Overall bias** | |  | | | Low |
| **PubMed ID** | 30617130 | | **First author** | Ramchandren R | |
| **Domain** | | **Assessment items** | | | **Risk of bias judgement** |
| **Bias arising from the randomization process** | | Was the randomization process appropriate, including generation and concealment of the allocation sequence, with no evidence of baseline imbalance? | | | Low |
| **Bias due to deviations from intended interventions** | | Were participants and personnel blinded, and were any deviations from intended interventions likely to affect outcomes or be unbalanced between groups, with appropriate analysis of assigned groups? | | | Low |
| **Bias due to missing outcome data** | | Was outcome data available for all, or nearly all, participants randomized, and if not, is it unlikely that missingness depended on the true outcome or biased the results? | | | Low |
| **Bias in measurement of the outcome** | | Was the outcome measured appropriately, consistently across groups, and without likely influence from assessors’ knowledge of intervention assignment? | | | Low |
| **Bias in selection of the reported result** | | Was the result analyzed following a pre-specified analysis plan, without selective reporting from multiple eligible outcome measurements or analyses? | | | Low |
| **Overall bias** | |  | | | Low |
| **PubMed ID** | 30522922 | | **First author** | Horwitz S | |
| **Domain** | | **Assessment items** | | | **Risk of bias judgement** |
| **Bias arising from the randomization process** | | Was the randomization process appropriate, including generation and concealment of the allocation sequence, with no evidence of baseline imbalance? | | | Low |
| **Bias due to deviations from intended interventions** | | Were participants and personnel blinded, and were any deviations from intended interventions likely to affect outcomes or be unbalanced between groups, with appropriate analysis of assigned groups? | | | Low |
| **Bias due to missing outcome data** | | Was outcome data available for all, or nearly all, participants randomized, and if not, is it unlikely that missingness depended on the true outcome or biased the results? | | | Low |
| **Bias in measurement of the outcome** | | Was the outcome measured appropriately, consistently across groups, and without likely influence from assessors’ knowledge of intervention assignment? | | | Low |
| **Bias in selection of the reported result** | | Was the result analyzed following a pre-specified analysis plan, without selective reporting from multiple eligible outcome measurements or analyses? | | | Low |
| **Overall bias** | |  | | | Low |
| **PubMed ID** | 31693429 | | **First author** | Sehn LH | |
| **Domain** | | **Assessment items** | | | **Risk of bias judgement** |
| **Bias arising from the randomization process** | | Was the randomization process appropriate, including generation and concealment of the allocation sequence, with no evidence of baseline imbalance? | | | Low |
| **Bias due to deviations from intended interventions** | | Were participants and personnel blinded, and were any deviations from intended interventions likely to affect outcomes or be unbalanced between groups, with appropriate analysis of assigned groups? | | | Low |
| **Bias due to missing outcome data** | | Was outcome data available for all, or nearly all, participants randomized, and if not, is it unlikely that missingness depended on the true outcome or biased the results? | | | Some concerns |
| **Bias in measurement of the outcome** | | Was the outcome measured appropriately, consistently across groups, and without likely influence from assessors’ knowledge of intervention assignment? | | | Some concerns |
| **Bias in selection of the reported result** | | Was the result analyzed following a pre-specified analysis plan, without selective reporting from multiple eligible outcome measurements or analyses? | | | Low |
| **Overall bias** | |  | | | Some concerns |
| **PubMed ID** | 30315239 | | **First author** | Fraser G | |
| **Domain** | | **Assessment items** | | | **Risk of bias judgement** |
| **Bias arising from the randomization process** | | Was the randomization process appropriate, including generation and concealment of the allocation sequence, with no evidence of baseline imbalance? | | | Low |
| **Bias due to deviations from intended interventions** | | Were participants and personnel blinded, and were any deviations from intended interventions likely to affect outcomes or be unbalanced between groups, with appropriate analysis of assigned groups? | | | Low |
| **Bias due to missing outcome data** | | Was outcome data available for all, or nearly all, participants randomized, and if not, is it unlikely that missingness depended on the true outcome or biased the results? | | | Low |
| **Bias in measurement of the outcome** | | Was the outcome measured appropriately, consistently across groups, and without likely influence from assessors’ knowledge of intervention assignment? | | | Low |
| **Bias in selection of the reported result** | | Was the result analyzed following a pre-specified analysis plan, without selective reporting from multiple eligible outcome measurements or analyses? | | | Low |
| **Overall bias** | |  | | | Low |
| **PubMed ID** | 31735560 | | **First author** | Attal M | |
| **Domain** | | **Assessment items** | | | **Risk of bias judgement** |
| **Bias arising from the randomization process** | | Was the randomization process appropriate, including generation and concealment of the allocation sequence, with no evidence of baseline imbalance? | | | Low |
| **Bias due to deviations from intended interventions** | | Were participants and personnel blinded, and were any deviations from intended interventions likely to affect outcomes or be unbalanced between groups, with appropriate analysis of assigned groups? | | | Low |
| **Bias due to missing outcome data** | | Was outcome data available for all, or nearly all, participants randomized, and if not, is it unlikely that missingness depended on the true outcome or biased the results? | | | Low |
| **Bias in measurement of the outcome** | | Was the outcome measured appropriately, consistently across groups, and without likely influence from assessors’ knowledge of intervention assignment? | | | Low |
| **Bias in selection of the reported result** | | Was the result analyzed following a pre-specified analysis plan, without selective reporting from multiple eligible outcome measurements or analyses? | | | Low |
| **Overall bias** | |  | | | Low |
| **PubMed ID** | 30522969 | | **First author** | Moreno C | |
| **Domain** | | **Assessment items** | | | **Risk of bias judgement** |
| **Bias arising from the randomization process** | | Was the randomization process appropriate, including generation and concealment of the allocation sequence, with no evidence of baseline imbalance? | | | Low |
| **Bias due to deviations from intended interventions** | | Were participants and personnel blinded, and were any deviations from intended interventions likely to affect outcomes or be unbalanced between groups, with appropriate analysis of assigned groups? | | | Low |
| **Bias due to missing outcome data** | | Was outcome data available for all, or nearly all, participants randomized, and if not, is it unlikely that missingness depended on the true outcome or biased the results? | | | Low |
| **Bias in measurement of the outcome** | | Was the outcome measured appropriately, consistently across groups, and without likely influence from assessors’ knowledge of intervention assignment? | | | Low |
| **Bias in selection of the reported result** | | Was the result analyzed following a pre-specified analysis plan, without selective reporting from multiple eligible outcome measurements or analyses? | | | Low |
| **Overall bias** | |  | | | Low |
| **PubMed ID** | 31801940 | | **First author** | van Oers M | |
| **Domain** | | **Assessment items** | | | **Risk of bias judgement** |
| **Bias arising from the randomization process** | | Was the randomization process appropriate, including generation and concealment of the allocation sequence, with no evidence of baseline imbalance? | | | Low |
| **Bias due to deviations from intended interventions** | | Were participants and personnel blinded, and were any deviations from intended interventions likely to affect outcomes or be unbalanced between groups, with appropriate analysis of assigned groups? | | | Low |
| **Bias due to missing outcome data** | | Was outcome data available for all, or nearly all, participants randomized, and if not, is it unlikely that missingness depended on the true outcome or biased the results? | | | Low |
| **Bias in measurement of the outcome** | | Was the outcome measured appropriately, consistently across groups, and without likely influence from assessors’ knowledge of intervention assignment? | | | Some concerns |
| **Bias in selection of the reported result** | | Was the result analyzed following a pre-specified analysis plan, without selective reporting from multiple eligible outcome measurements or analyses? | | | Low |
| **Overall bias** | |  | | | Some concerns |
| **PubMed ID** | 32459600 | | **First author** | Ghia P | |
| **Domain** | | **Assessment items** | | | **Risk of bias judgement** |
| **Bias arising from the randomization process** | | Was the randomization process appropriate, including generation and concealment of the allocation sequence, with no evidence of baseline imbalance? | | | Low |
| **Bias due to deviations from intended interventions** | | Were participants and personnel blinded, and were any deviations from intended interventions likely to affect outcomes or be unbalanced between groups, with appropriate analysis of assigned groups? | | | Some concerns |
| **Bias due to missing outcome data** | | Was outcome data available for all, or nearly all, participants randomized, and if not, is it unlikely that missingness depended on the true outcome or biased the results? | | | Low |
| **Bias in measurement of the outcome** | | Was the outcome measured appropriately, consistently across groups, and without likely influence from assessors’ knowledge of intervention assignment? | | | Low |
| **Bias in selection of the reported result** | | Was the result analyzed following a pre-specified analysis plan, without selective reporting from multiple eligible outcome measurements or analyses? | | | Low |
| **Overall bias** | |  | | | Some concerns |
| **PubMed ID** | 32305093 | | **First author** | Sharman JP | |
| **Domain** | | **Assessment items** | | | **Risk of bias judgement** |
| **Bias arising from the randomization process** | | Was the randomization process appropriate, including generation and concealment of the allocation sequence, with no evidence of baseline imbalance? | | | Low |
| **Bias due to deviations from intended interventions** | | Were participants and personnel blinded, and were any deviations from intended interventions likely to affect outcomes or be unbalanced between groups, with appropriate analysis of assigned groups? | | | Low |
| **Bias due to missing outcome data** | | Was outcome data available for all, or nearly all, participants randomized, and if not, is it unlikely that missingness depended on the true outcome or biased the results? | | | Low |
| **Bias in measurement of the outcome** | | Was the outcome measured appropriately, consistently across groups, and without likely influence from assessors’ knowledge of intervention assignment? | | | Low |
| **Bias in selection of the reported result** | | Was the result analyzed following a pre-specified analysis plan, without selective reporting from multiple eligible outcome measurements or analyses? | | | Low |
| **Overall bias** | |  | | | Low |
| **PubMed ID** | 32810220 | | **First author** | Maloney DG | |
| **Domain** | | **Assessment items** | | | **Risk of bias judgement** |
| **Bias arising from the randomization process** | | Was the randomization process appropriate, including generation and concealment of the allocation sequence, with no evidence of baseline imbalance? | | | Low |
| **Bias due to deviations from intended interventions** | | Were participants and personnel blinded, and were any deviations from intended interventions likely to affect outcomes or be unbalanced between groups, with appropriate analysis of assigned groups? | | | Low |
| **Bias due to missing outcome data** | | Was outcome data available for all, or nearly all, participants randomized, and if not, is it unlikely that missingness depended on the true outcome or biased the results? | | | Some concerns |
| **Bias in measurement of the outcome** | | Was the outcome measured appropriately, consistently across groups, and without likely influence from assessors’ knowledge of intervention assignment? | | | Some concerns |
| **Bias in selection of the reported result** | | Was the result analyzed following a pre-specified analysis plan, without selective reporting from multiple eligible outcome measurements or analyses? | | | Low |
| **Overall bias** | |  | | | Some concerns |
| **PubMed ID** | 31879945 | | **First author** | Pettengell R | |
| **Domain** | | **Assessment items** | | | **Risk of bias judgement** |
| **Bias arising from the randomization process** | | Was the randomization process appropriate, including generation and concealment of the allocation sequence, with no evidence of baseline imbalance? | | | Low |
| **Bias due to deviations from intended interventions** | | Were participants and personnel blinded, and were any deviations from intended interventions likely to affect outcomes or be unbalanced between groups, with appropriate analysis of assigned groups? | | | Low |
| **Bias due to missing outcome data** | | Was outcome data available for all, or nearly all, participants randomized, and if not, is it unlikely that missingness depended on the true outcome or biased the results? | | | Low |
| **Bias in measurement of the outcome** | | Was the outcome measured appropriately, consistently across groups, and without likely influence from assessors’ knowledge of intervention assignment? | | | Some concerns |
| **Bias in selection of the reported result** | | Was the result analyzed following a pre-specified analysis plan, without selective reporting from multiple eligible outcome measurements or analyses? | | | Low |
| **Overall bias** | |  | | | Some concerns |
| **PubMed ID** | 34097854 | | **First author** | Moreau P | |
| **Domain** | | **Assessment items** | | | **Risk of bias judgement** |
| **Bias arising from the randomization process** | | Was the randomization process appropriate, including generation and concealment of the allocation sequence, with no evidence of baseline imbalance? | | | Low |
| **Bias due to deviations from intended interventions** | | Were participants and personnel blinded, and were any deviations from intended interventions likely to affect outcomes or be unbalanced between groups, with appropriate analysis of assigned groups? | | | Low |
| **Bias due to missing outcome data** | | Was outcome data available for all, or nearly all, participants randomized, and if not, is it unlikely that missingness depended on the true outcome or biased the results? | | | Low |
| **Bias in measurement of the outcome** | | Was the outcome measured appropriately, consistently across groups, and without likely influence from assessors’ knowledge of intervention assignment? | | | Low |
| **Bias in selection of the reported result** | | Was the result analyzed following a pre-specified analysis plan, without selective reporting from multiple eligible outcome measurements or analyses? | | | Low |
| **Overall bias** | |  | | | Low |
| **PubMed ID** | 34843406 | | **First author** | Bachy E | |
| **Domain** | | **Assessment items** | | | **Risk of bias judgement** |
| **Bias arising from the randomization process** | | Was the randomization process appropriate, including generation and concealment of the allocation sequence, with no evidence of baseline imbalance? | | | Low |
| **Bias due to deviations from intended interventions** | | Were participants and personnel blinded, and were any deviations from intended interventions likely to affect outcomes or be unbalanced between groups, with appropriate analysis of assigned groups? | | | Some concerns |
| **Bias due to missing outcome data** | | Was outcome data available for all, or nearly all, participants randomized, and if not, is it unlikely that missingness depended on the true outcome or biased the results? | | | Low |
| **Bias in measurement of the outcome** | | Was the outcome measured appropriately, consistently across groups, and without likely influence from assessors’ knowledge of intervention assignment? | | | Low |
| **Bias in selection of the reported result** | | Was the result analyzed following a pre-specified analysis plan, without selective reporting from multiple eligible outcome measurements or analyses? | | | Low |
| **Overall bias** | |  | | | Some concerns |
| **PubMed ID** | 34310172 | | **First author** | Byrd JC | |
| **Domain** | | **Assessment items** | | | **Risk of bias judgement** |
| **Bias arising from the randomization process** | | Was the randomization process appropriate, including generation and concealment of the allocation sequence, with no evidence of baseline imbalance? | | | Low |
| **Bias due to deviations from intended interventions** | | Were participants and personnel blinded, and were any deviations from intended interventions likely to affect outcomes or be unbalanced between groups, with appropriate analysis of assigned groups? | | | Some concerns |
| **Bias due to missing outcome data** | | Was outcome data available for all, or nearly all, participants randomized, and if not, is it unlikely that missingness depended on the true outcome or biased the results? | | | Low |
| **Bias in measurement of the outcome** | | Was the outcome measured appropriately, consistently across groups, and without likely influence from assessors’ knowledge of intervention assignment? | | | Low |
| **Bias in selection of the reported result** | | Was the result analyzed following a pre-specified analysis plan, without selective reporting from multiple eligible outcome measurements or analyses? | | | Low |
| **Overall bias** | |  | | | Some concerns |
| **PubMed ID** | 33763699 | | **First author** | Facon T | |
| **Domain** | | **Assessment items** | | | **Risk of bias judgement** |
| **Bias arising from the randomization process** | | Was the randomization process appropriate, including generation and concealment of the allocation sequence, with no evidence of baseline imbalance? | | | Low |
| **Bias due to deviations from intended interventions** | | Were participants and personnel blinded, and were any deviations from intended interventions likely to affect outcomes or be unbalanced between groups, with appropriate analysis of assigned groups? | | | Low |
| **Bias due to missing outcome data** | | Was outcome data available for all, or nearly all, participants randomized, and if not, is it unlikely that missingness depended on the true outcome or biased the results? | | | Low |
| **Bias in measurement of the outcome** | | Was the outcome measured appropriately, consistently across groups, and without likely influence from assessors’ knowledge of intervention assignment? | | | Low |
| **Bias in selection of the reported result** | | Was the result analyzed following a pre-specified analysis plan, without selective reporting from multiple eligible outcome measurements or analyses? | | | Low |
| **Overall bias** | |  | | | Low |
| **PubMed ID** | 38319255 | | **First author** | Kater AP | |
| **Domain** | | **Assessment items** | | | **Risk of bias judgement** |
| **Bias arising from the randomization process** | | Was the randomization process appropriate, including generation and concealment of the allocation sequence, with no evidence of baseline imbalance? | | | Low |
| **Bias due to deviations from intended interventions** | | Were participants and personnel blinded, and were any deviations from intended interventions likely to affect outcomes or be unbalanced between groups, with appropriate analysis of assigned groups? | | | Low |
| **Bias due to missing outcome data** | | Was outcome data available for all, or nearly all, participants randomized, and if not, is it unlikely that missingness depended on the true outcome or biased the results? | | | Low |
| **Bias in measurement of the outcome** | | Was the outcome measured appropriately, consistently across groups, and without likely influence from assessors’ knowledge of intervention assignment? | | | Low |
| **Bias in selection of the reported result** | | Was the result analyzed following a pre-specified analysis plan, without selective reporting from multiple eligible outcome measurements or analyses? | | | Low |
| **Overall bias** | |  | | | Low |
| **PubMed ID** | 35810754 | | **First author** | Tam CS | |
| **Domain** | | **Assessment items** | | | **Risk of bias judgement** |
| **Bias arising from the randomization process** | | Was the randomization process appropriate, including generation and concealment of the allocation sequence, with no evidence of baseline imbalance? | | | Low |
| **Bias due to deviations from intended interventions** | | Were participants and personnel blinded, and were any deviations from intended interventions likely to affect outcomes or be unbalanced between groups, with appropriate analysis of assigned groups? | | | Some concerns |
| **Bias due to missing outcome data** | | Was outcome data available for all, or nearly all, participants randomized, and if not, is it unlikely that missingness depended on the true outcome or biased the results? | | | Low |
| **Bias in measurement of the outcome** | | Was the outcome measured appropriately, consistently across groups, and without likely influence from assessors’ knowledge of intervention assignment? | | | Low |
| **Bias in selection of the reported result** | | Was the result analyzed following a pre-specified analysis plan, without selective reporting from multiple eligible outcome measurements or analyses? | | | Low |
| **Overall bias** | |  | | | Some concerns |
| **PubMed ID** | 35032434 | | **First author** | Schjesvold FH | |
| **Domain** | | **Assessment items** | | | **Risk of bias judgement** |
| **Bias arising from the randomization process** | | Was the randomization process appropriate, including generation and concealment of the allocation sequence, with no evidence of baseline imbalance? | | | Low |
| **Bias due to deviations from intended interventions** | | Were participants and personnel blinded, and were any deviations from intended interventions likely to affect outcomes or be unbalanced between groups, with appropriate analysis of assigned groups? | | | Low |
| **Bias due to missing outcome data** | | Was outcome data available for all, or nearly all, participants randomized, and if not, is it unlikely that missingness depended on the true outcome or biased the results? | | | Low |
| **Bias in measurement of the outcome** | | Was the outcome measured appropriately, consistently across groups, and without likely influence from assessors’ knowledge of intervention assignment? | | | Low |
| **Bias in selection of the reported result** | | Was the result analyzed following a pre-specified analysis plan, without selective reporting from multiple eligible outcome measurements or analyses? | | | Low |
| **Overall bias** | |  | | | Low |
| **PubMed ID** | 36511784 | | **First author** | Brown JR | |
| **Domain** | | **Assessment items** | | | **Risk of bias judgement** |
| **Bias arising from the randomization process** | | Was the randomization process appropriate, including generation and concealment of the allocation sequence, with no evidence of baseline imbalance? | | | Low |
| **Bias due to deviations from intended interventions** | | Were participants and personnel blinded, and were any deviations from intended interventions likely to affect outcomes or be unbalanced between groups, with appropriate analysis of assigned groups? | | | Some concerns |
| **Bias due to missing outcome data** | | Was outcome data available for all, or nearly all, participants randomized, and if not, is it unlikely that missingness depended on the true outcome or biased the results? | | | Low |
| **Bias in measurement of the outcome** | | Was the outcome measured appropriately, consistently across groups, and without likely influence from assessors’ knowledge of intervention assignment? | | | Some concerns |
| **Bias in selection of the reported result** | | Was the result analyzed following a pre-specified analysis plan, without selective reporting from multiple eligible outcome measurements or analyses? | | | Low |
| **Overall bias** | |  | | | Some concerns |
| **PubMed ID** | 37404773 | | **First author** | Townsend W | |
| **Domain** | | **Assessment items** | | | **Risk of bias judgement** |
| **Bias arising from the randomization process** | | Was the randomization process appropriate, including generation and concealment of the allocation sequence, with no evidence of baseline imbalance? | | | Low |
| **Bias due to deviations from intended interventions** | | Were participants and personnel blinded, and were any deviations from intended interventions likely to affect outcomes or be unbalanced between groups, with appropriate analysis of assigned groups? | | | Some concerns |
| **Bias due to missing outcome data** | | Was outcome data available for all, or nearly all, participants randomized, and if not, is it unlikely that missingness depended on the true outcome or biased the results? | | | Low |
| **Bias in measurement of the outcome** | | Was the outcome measured appropriately, consistently across groups, and without likely influence from assessors’ knowledge of intervention assignment? | | | Some concerns |
| **Bias in selection of the reported result** | | Was the result analyzed following a pre-specified analysis plan, without selective reporting from multiple eligible outcome measurements or analyses? | | | Low |
| **Overall bias** | |  | | | Some concerns |
| **PubMed ID** | 37506346 | | **First author** | Zinzani PL | |
| **Domain** | | **Assessment items** | | | **Risk of bias judgement** |
| **Bias arising from the randomization process** | | Was the randomization process appropriate, including generation and concealment of the allocation sequence, with no evidence of baseline imbalance? | | | Low |
| **Bias due to deviations from intended interventions** | | Were participants and personnel blinded, and were any deviations from intended interventions likely to affect outcomes or be unbalanced between groups, with appropriate analysis of assigned groups? | | | Some concerns |
| **Bias due to missing outcome data** | | Was outcome data available for all, or nearly all, participants randomized, and if not, is it unlikely that missingness depended on the true outcome or biased the results? | | | Low |
| **Bias in measurement of the outcome** | | Was the outcome measured appropriately, consistently across groups, and without likely influence from assessors’ knowledge of intervention assignment? | | | Low |
| **Bias in selection of the reported result** | | Was the result analyzed following a pre-specified analysis plan, without selective reporting from multiple eligible outcome measurements or analyses? | | | Low |
| **Overall bias** | |  | | | Some concerns |
| **PubMed ID** | 38796193 | | **First author** | Dupuis J | |
| **Domain** | | **Assessment items** | | | **Risk of bias judgement** |
| **Bias arising from the randomization process** | | Was the randomization process appropriate, including generation and concealment of the allocation sequence, with no evidence of baseline imbalance? | | | Low |
| **Bias due to deviations from intended interventions** | | Were participants and personnel blinded, and were any deviations from intended interventions likely to affect outcomes or be unbalanced between groups, with appropriate analysis of assigned groups? | | | Some concerns |
| **Bias due to missing outcome data** | | Was outcome data available for all, or nearly all, participants randomized, and if not, is it unlikely that missingness depended on the true outcome or biased the results? | | | Low |
| **Bias in measurement of the outcome** | | Was the outcome measured appropriately, consistently across groups, and without likely influence from assessors’ knowledge of intervention assignment? | | | Some concerns |
| **Bias in selection of the reported result** | | Was the result analyzed following a pre-specified analysis plan, without selective reporting from multiple eligible outcome measurements or analyses? | | | Low |
| **Overall bias** | |  | | | Some concerns |
